# Correlation between Oncogenic Mutations and Parameter Sensitivity of the Apoptosis Pathway Model

**DOI:** 10.1371/journal.pcbi.1003451

**Published:** 2014-01-23

**Authors:** Jia Chen, Haicen Yue, Qi Ouyang

**Affiliations:** 1 Center for Quantitative Biology and Peking-Tsinghua Center for Life Sciences at Peking University, Beijing, China; 2 School of Physics and the State Key Laboratory for Artificial Microstructures and Mesoscopic Physics, Peking University, Beijing, China; University of California, San Diego, United States of America

## Abstract

One of the major breakthroughs in oncogenesis research in recent years is the discovery that, in most patients, oncogenic mutations are concentrated in a few core biological functional pathways. This discovery indicates that oncogenic mechanisms are highly related to the dynamics of biologic regulatory networks, which govern the behaviour of functional pathways. Here, we propose that oncogenic mutations found in different biological functional pathways are closely related to parameter sensitivity of the corresponding networks. To test this hypothesis, we focus on the DNA damage-induced apoptotic pathway—the most important safeguard against oncogenesis. We first built the regulatory network that governs the apoptosis pathway, and then translated the network into dynamics equations. Using sensitivity analysis of the network parameters and comparing the results with cancer gene mutation spectra, we found that parameters that significantly affect the bifurcation point correspond to high-frequency oncogenic mutations. This result shows that the position of the bifurcation point is a better measure of the functionality of a biological network than gene expression levels of certain key proteins. It further demonstrates the suitability of applying systems-level analysis to biological networks as opposed to studying genes or proteins in isolation.

## Author Summary

Among complex genetic diseases affecting humans, cancer is a major cause of death. In 2008, a genome-wide analysis of hundreds of tumour samples showed that oncogenic mutations are concentrated in a few core functional pathways, revealing a new conceptual framework for cancer biology research, where the role of oncogenic mutations and oncogenic mechanisms are addressed from a network perspective. We therefore propose a new way of identifying high-frequency gene mutations in cancer: gene mutations may affect their corresponding proteins' activity in the biological regulatory network and can be considered as perturbations of the dynamical system. Therefore, mutations that induce qualitative changes in biological networks should correspond to high-frequency mutations in cancer. This concept can help us identify and understand the function of genes that play an important role in oncogenesis, thereby allowing targeted and effective design of gene-based therapy in cancer.

## Introduction

Cancer is one of the most important diseases affecting human health today [1]. Although cancer is considered a genetic disease [2], with a variety of oncogenes and tumour suppressor genes identified, the specific genomic alterations vary wildly between and within cancer types. In 2008, three high-throughput cancer genomic studies reported that cancer gene mutations are concentrated in a limited number of core cellular pathways and regulatory processes [3]–[5]. This discovery suggests that oncogenesis is highly related to the dynamics of biologic regulatory networks, which govern the behaviour of functional pathways. Clearly, to understand the mechanisms underlying oncogenesis, we need to take a systems and dynamics approach.

A number of studies have proposed a network-based approach to investigate oncogenesis. For example, Torkamani and Schork identified functionally related gene modules targeted by somatic mutation in cancer [6]; Cerami et al. proposed an automated network analysis approach to identify candidate oncogenic processes [7]. A more recent approach by Stites et al. sought to explain mutations in Ras pathway, which are commonly found in cancer, by investigating the steady state concentrations of cellular proteins in parameters changes [8].

In this paper, we propose a new way to identify high-frequency gene mutations in cancer cells. We reason that because gene mutations may affect the activities of their corresponding proteins in a biological regulatory network, they can be considered as perturbations of the system's dynamics. Therefore, those mutations that qualitatively affect biological network function should correspond to mutation hot spots in cancer. From a dynamics point of view, a qualitative change in a system relates to bifurcations—oncogenic mutations should therefore significantly affect certain bifurcation points.

One of the hallmarks of cancer is evasion of apoptosis; in fact p53 mutations are found in most human cancers [9]. We therefore chose the DNA damage-induced p53-centered apoptosis pathway, as an example, to evaluate our hypothesis. We evaluated the sensitivity of bifurcation points to different network parameters, and compared the results with the cancer gene mutation spectrum. We found that parameters that significantly affect the bifurcation points corresponded to high-frequency oncogenic mutations. This study investigates the mutation spectrum found in cancer cells and provides a useful tool for predicting oncogenic mutations.

## Results

### Network description and model building

We focused on the apoptotic pathway that responds to sustained DNA damage, induced by the chemotherapeutic compound, etoposide [10], [11]. A recent study showed that while low-dose etoposide induces oscillations in p53 levels, caspase3 levels remain low, and most cells survive; in contrast, high-dose etoposide induces a monotonic increase in p53 concentration, followed by a rapid increase in caspase3 with most cells undergoing apoptosis [11]. This experiment further justifies the use of p53 in our model.

**Figure 1:**
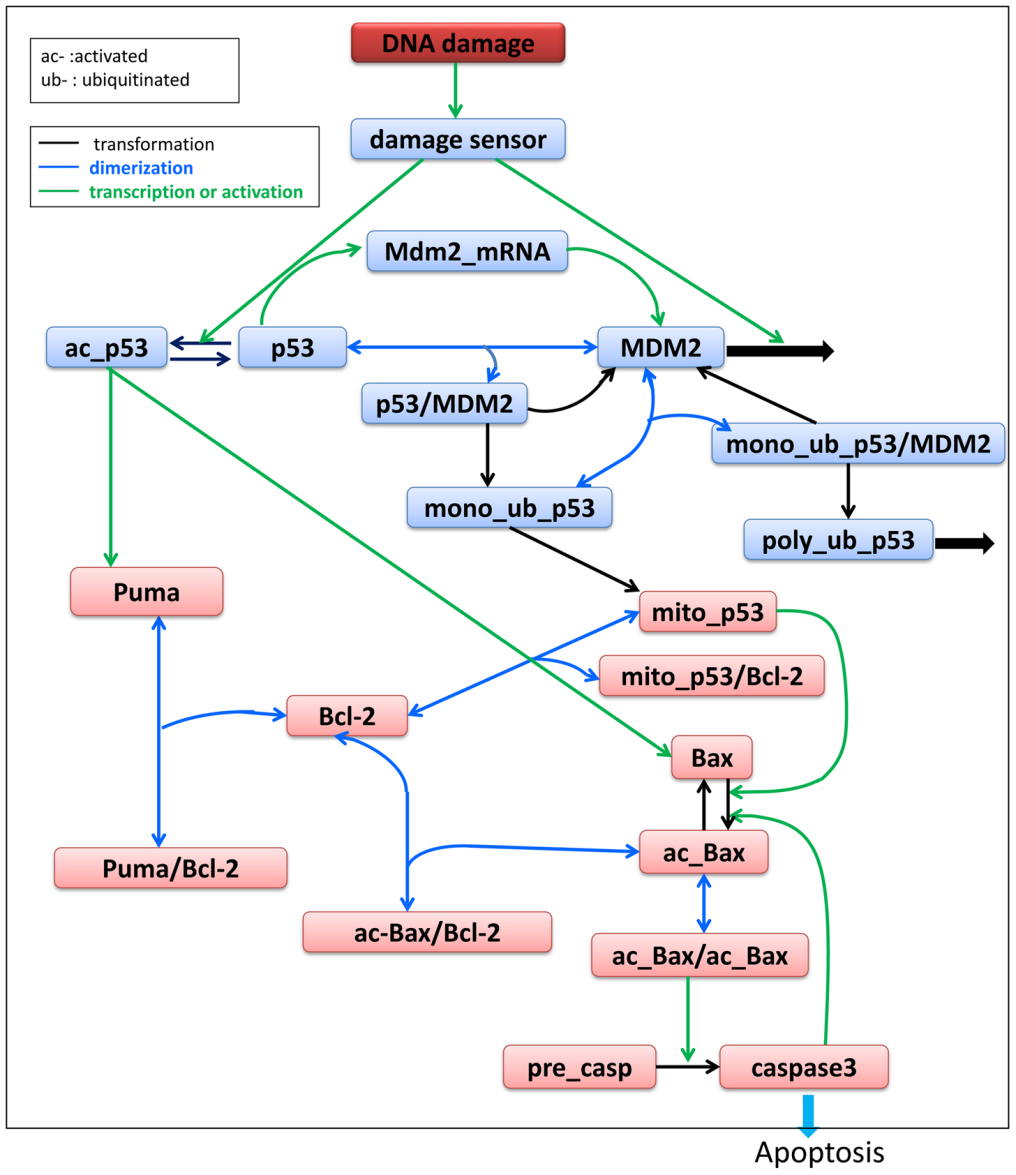
DNA damage-induced apoptotic pathway. Blue lines indicate dimerization; black lines indicate transformation; green lines indicate transcription or activation. “mono-ub”, mono-ubiquitinated; “poly-ub”, poly-ubiquitinated; “mito-” mitochondrial and “/” refers to a complex. The production and degradation of most components are not drawn but are included in the ODEs. Protein families with similar functions are grouped into one node/variable denoted by their representative members (e.g. Bax stands for Bax and Bak). High and low levels of caspase-3 indicate apoptosis and survival, respectively.

A schematic of the corresponding regulatory network, which is a modification of the p53 DNA damage response network established and analysed by Li et al. [12], is shown in Figure 1. Nuclear p53 induces *mdm2* transcription, while MDM2 antagonizes p53 by promoting multistep ubiquitination and proteasome-dependent degradation of p53 [13], [14]. In unstressed cells, p53 is kept at a low concentration by its negative regulator MDM2. DNA damage reduces the binding affinity between p53 and MDM2 by inducing phosphorylation of p53 and MDM2 [15] — phosphorylated MDM2 undergoes rapid degradation [16] and p53 is subsequently activated by phosphorylation to a “response state”, triggering downstream events, such as apoptosis and cell-cycle arrest [17]. As shown in Figure 1, mono-ubiquitinated p53 is exported to the cytoplasm, while poly-ubiquitinated p53 undergoes degradation [18]. At elevated p53 levels, apoptosis can be initiated by both nuclear and cytoplasmic (or mitochondrial) p53 [19]. While nuclear p53 regulates the transcription of pro-apoptotic proteins such as Puma, Noxa, Bax and Bak [20], mitochondrial p53 exerts a direct pro-apoptotic effect by interacting with Bax and Bak to form a positive feedback loop that activates caspase3 [20]–[24]. In the experiments conducted by Chipuk et al. [21] and Chen et al. [11], p53 appears to regulate apoptosis through its cytoplasmic pro-death activity and not its nuclear activity.

Many previous studies of p53 dynamics have focused on the response to transient DNA damage induced by ionising radiation or UV; following cell-cycle arrest, cell proliferation resumes once the DNA damage is repaired [25], [26]. In our model, we consider sustained DNA damage, which is maintained at a constant damage level until the cell initiates apoptosis [12]. In this way, we can ignore the cell-cycle arrest pathway, and instead concentrate on the apoptosis pathway.

### Model simulation

**Figure 2:**
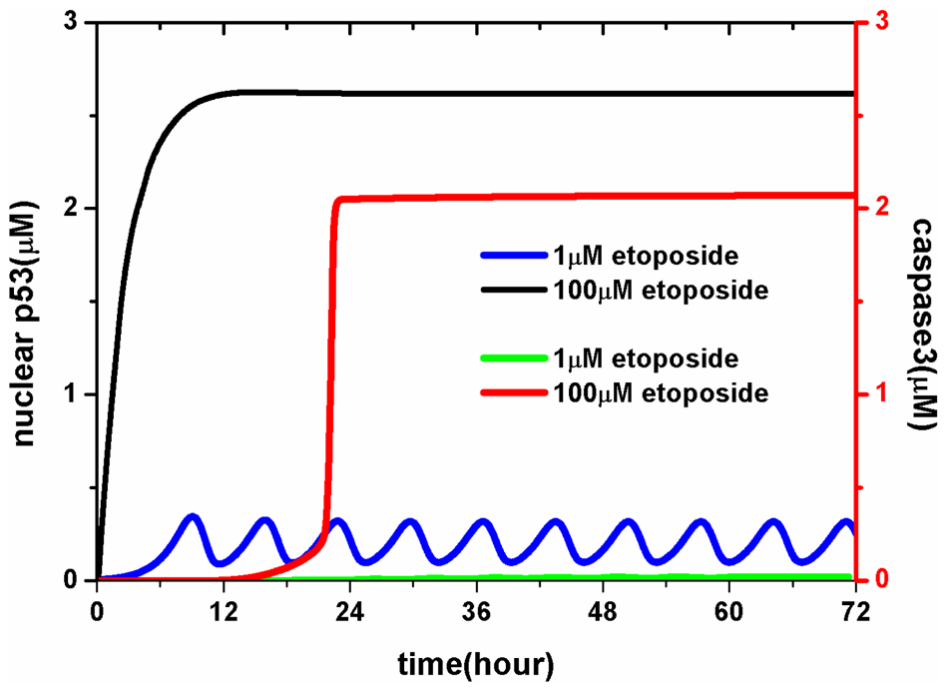
The typical time evolution of the level of total nuclear p53 and caspase3. Blue and black lines represent p53 concentrations at low- and high-level DNA damage, respectively; Green and red lines represent caspase-3 concentrations at low- and high-level DNA damage, respectively.

We first present an overview of network dynamics in response to DNA damage at two typical doses of etoposide. As shown in Figure 2, at 1 µM etoposide (low DNA damage), the concentration of nuclear p53 (including inactivated- and activated-p53) oscillates around basal levels (blue line), while the concentration of caspase3 remains low (green line), indicating that apoptosis has not been trigged. At 100 µM etoposide (high DNA damage), nuclear p53 increases monotonically (black line), which is followed by a rapid increase in caspase3 (red line), which in turn triggers downstream apoptotic processes. These results are consistent with the experimental observations of Chen et al. [11], indicating that our model qualitatively reflects the real system.

**Figure 3:**
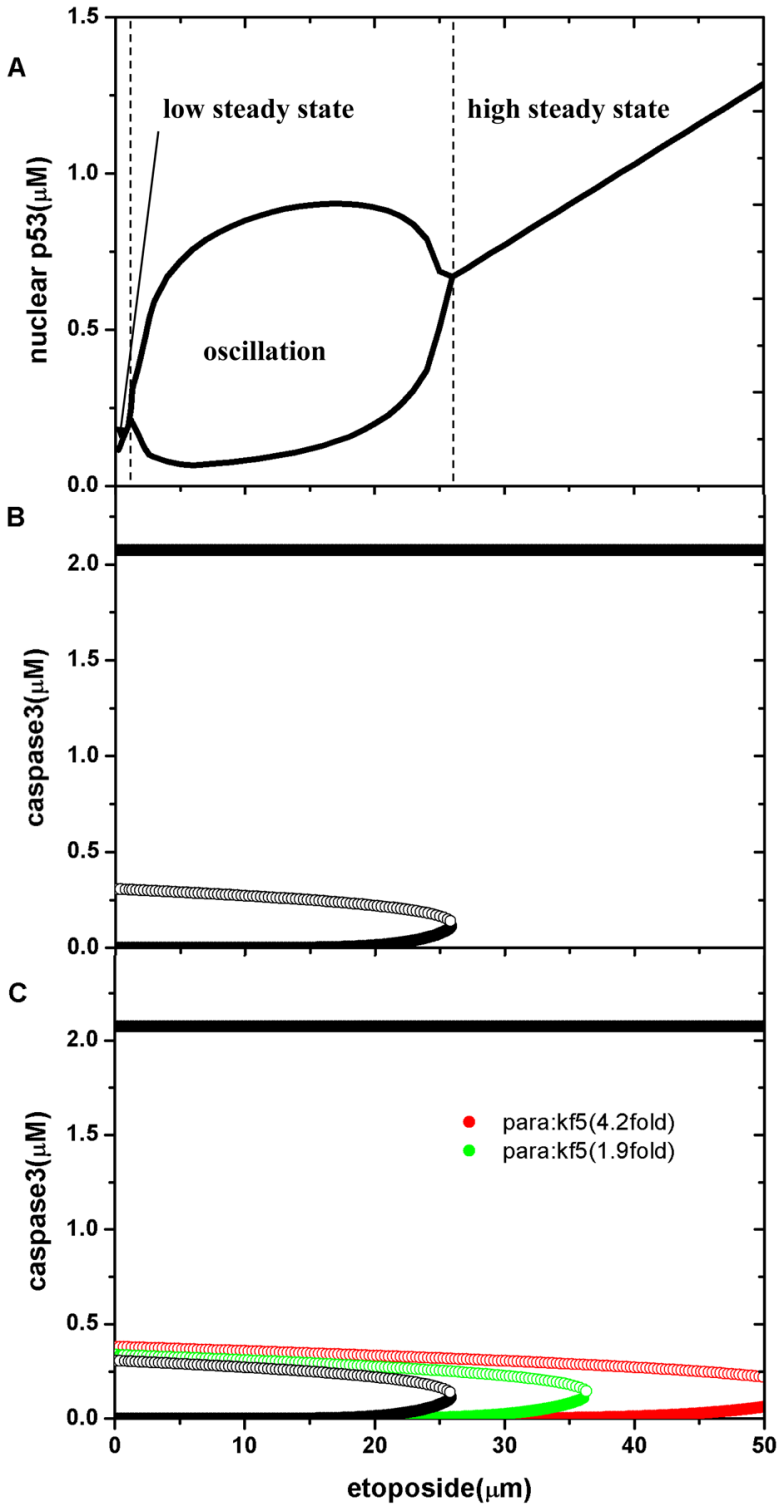
Bifurcation diagram for nuclear p53 and caspase3 for apoptosis. (A) The bifurcation diagram for nuclear p53 using DNA damage as the control parameter. (B) The bifurcation diagram for caspase3 using DNA damage as the control parameter (black dots). (C) Comparison of bifurcation point with an increase in the parameter kf5. Black, without increase; green, 1.9-fold increase; red, 4.2-fold increase.

Having created a suitable model, we next conducted bifurcation analysis to identify potential qualitative changes in the system. Using the level of DNA damage as the control parameter, two types of bifurcations were found in this analysis: two Hopf bifurcations and one saddle-node bifurcation. The transition diagrams of these bifurcations are presented in Figure 3. In Figure 3A, as the level of DNA damage increases, nuclear p53 undergoes two Hopf bifurcations. In the first bifurcation, with increasing DNA damage, the system changes from a low steady state to an oscillatory state; in the second bifurcation, with further DNA damage, the oscillatory state changes to a high steady state. This result is consistent with previously published observations [10], [12], [27]–[29]. In the case of caspase3, there exists a saddle-node bifurcation as a function of the level of DNA damage, where a stable node collides with an unstable saddle at the bifurcation point, as shown in Figure 3B. This bifurcation separates the system into two dynamic regimes: a mono-stable steady state regime and a bistable regime. In the bistable regime, one steady state corresponds to a low caspase3 concentration and the other to a high caspase3 concentration. In a wild-type cell, the caspase3 concentration remains low. Once the DNA damage level increases beyond the bifurcation point (about 26 µM etoposide in Figure 3B), the system will switch to a high caspase3 concentration that turns on the apoptosis pathway. Notice that this switch is irreversible—once the apoptosis pathway is turned on, caspase3 can maintain the high level state even when the level of DNA damage falls below the initial threshold.

Our model is based on biological facts, together with certain assumptions and simplifications. The details of our model are presented in the Supporting Information (Text S1). Notice that in our model, ac-p53 refers to phosphorylated p53.

### Parameter sensitivity

In biological systems, many biological functions are controlled through dynamic bifurcations. A good example is the saddle-node bifurcation in G1/S transition of the cell cycle [30]–[33], where the qualitative behaviour of the system is significantly affected by changes in control parameters, which may dramatically affect the location of the critical point. Figure 3C represents such an example in the apoptosis pathway, where increasing the control parameter kf5 (which corresponds to the rate of association of mono-ub-p53 and MDM2 in the regulatory network of Figure 1) 1.9-fold, shifts the critical point to the right. If kf5 is increased 4.2-fold, the high caspase3 state can never be reached under medium or high drug dose. This indicates that if the parameter kf5 is increased due to certain mutations, apoptosis will not be initiated properly, or will not initiate at all, even when the DNA is seriously damaged. The damaged cells therefore have a chance to bypass apoptosis, which may facilitate oncogenesis.

The effect of parameter changes on the location of the bifurcation point is not evenly distributed: some parameters significantly impact the bifurcation points, while others do not. We believe that genes that fall in the former category play important roles in oncogenesis.

To identify which parameters have a major impact on the location of the bifurcation point, we conducted parameter sensitivity analysis by increasing and decreasing each of the 54 parameters in our model 1.2-fold and recording the percentage change of the bifurcation points. In this way, we established a spectrum of parameter sensitivity, which allowed us to compare the result with the oncogenic mutation spectrum.

### The gene mutation spectrum of cancer

High-throughput cancer genomic projects, such as the Cancer Genome Atlas (TCGA) and the Catalogue of Somatic Mutations in Cancers (COSMIC) [34], are major resources to obtain the spectrum of genetic variants in different cancer types [35]. To investigate the relationship between parameter changes and the spectrum of cancer gene mutations, we chose skin cancer mutated genes from the Catalogue of Somatic Mutations in Cancers (COSMIC) and glioblastoma multiforme mutated genes from the “CAN-genes” by Parsons et al. [4], and TCGA [5].

Based on the knowledge of biochemical reactions and gene expression [3]–[5], we concentrated on three types of gene mutations: somatic mutations, amplifications, and deletions. Each mutation corresponds to specific parameters in ordinary differential equations (ODEs) in our model, the basis and details of which are given in the Supporting Information (Text S1).

### The correspondence between parameter sensitivity and mutated genes

**Figure 4:**
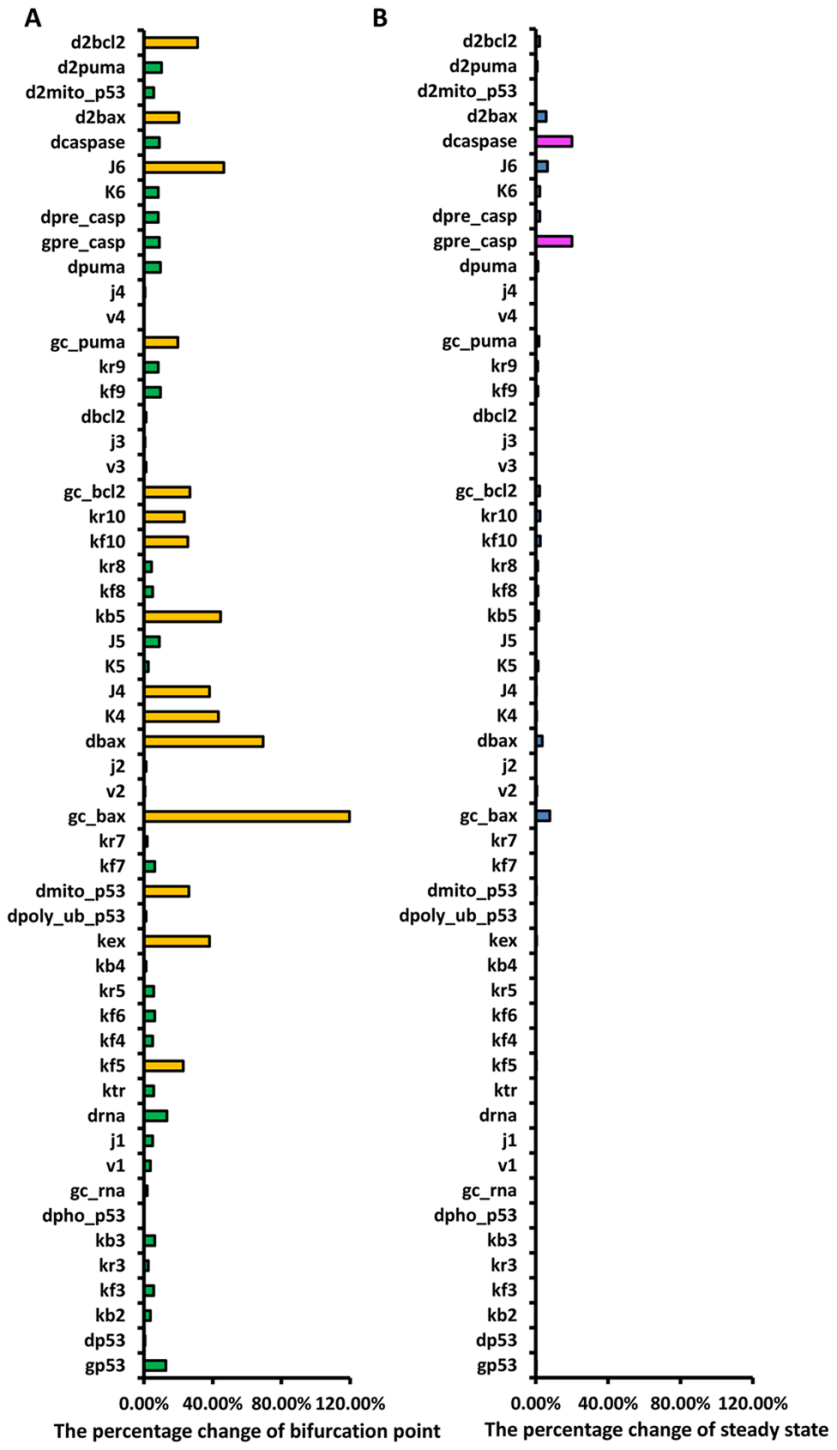
Parameter sensitivity analysis. (A) The change of the saddle-node bifurcation point in response to a 20% increase or decrease in each parameter of the apoptosis pathway. (B) The change in the steady-state concentration of caspase3 in response to a 20% increase or decrease in each parameter of the apoptosis pathway.

The main result of our calculation is summarized in Figure 4. Parameter sensitivities of the saddle-node bifurcation point of each parameter of the apoptosis pathway are shown in Figure 4A (see also Supporting Information Table S2); parameters that cause large or small changes in bifurcation points are marked in yellow, and green, respectively. For the apoptosis pathway about 70% of the parameters are non-influential: the bifurcation point varies very little when changing those parameters. This suggests that the apoptosis pathway is robust, a hallmark of biological networks. However, about 26% of parameters have significant effects on the critical bifurcation points, such as gc_Bax (the basal generation rate of Bax) and kex (nuclear-export rate of mono-ubiquitinated p53), see Figure 4A. A small change in these parameters will induce large changes in the bifurcation points, as shown in Figure 3B. Increasing gc_Bax causes the critical bifurcation point to shift to the left. Therefore, in a biological experiment, to achieve a given rate of apoptosis, increasing the basal generation rate of Bax will require a lower dose of the DNA damaging drug compared with the unaltered basal generation rate of Bax. Similarly, increasing kex will cause the critical bifurcation point to shift to the left, so that for a given rate of apoptosis, increasing the nuclear-export rate of mono-ubiquitinated p53 will require a lower dose of the DNA damaging drug compared with the unaltered nuclear-export rate.

Our parameter sensitivity analysis of critical bifurcation points is in agreement with the literature. For example, overexpression of Bax significantly increases the rate of radiation-induced apoptosis in human breast cancer cells [36], indicating that a perturbation of the basal generation rate of Bax (in our model the corresponding parameter is gc_Bax) would significantly affect the rate of apoptosis—as is indeed predicted by our model. Furthermore, mitochondrial p53-translocation and -accumulation may be induced by a variety of apoptotic stimuli, [37],[38]. In fact Marchenko et al. found that the rate of apoptosis is significantly increased after redirecting p53 from the nucleus to the mitochondria by using mitochondrial import leader peptides [37]. This means that a perturbation in the nuclear-export rate of p53 (in our model the corresponding parameter is kex) could greatly alter the rate of apoptosis. Again, we confirmed this effect in our model.

Moreover, Dewson et al. recently reported that following apoptotic signalling in cells and mitochondrial fractions, Bax homodimerises via a BH3:groove interface interaction [39], a necessary step in the apoptotic pathway. The key interaction domains that affect apoptotic function are located in the α2–α5 regions (54–126A) of Bax; mutations in one of these key residues disrupt apoptotic function, thereby reducing the rate of cell death following treatment with etoposide [39]. According to the COSMIC database, cancer mutation hot spots do exist in the α2–α5 helices of Bax. These loss-of-function mutations in the Bax BH3 domain decrease the dimerization rate of activated Bax (in our model the corresponding parameter is kf10). Our model showed that kf10 is indeed a sensitive parameter and a slight decrease in kf10 shifts the critical bifurcation point to the right.

We next sought to compare our results to those of Stites et al. who investigated mutations in Ras pathway by measuring the steady state concentrations of cellular proteins in parameter changes [8]. In addition to parameter sensitivity analysis of the bifurcation points, we therefore used the steady-state concentration of caspase3 as a measure of oncogenesis. To achieve this, we increased and decreased each of the 54 parameters 1.2-fold and recorded the percentage change in the steady-state concentration of caspase3. The results are presented in Figure 4B (See also Supporting Information Table S3). The parameters that cause a large or small percentage change in the steady-state concentration of caspase3 are marked in magenta and blue, respectively. Overall, we found that the bifurcation point and the steady-state concentration of caspase3 are sensitive to mutually exclusive sets of parameters.

**Figure 5:**
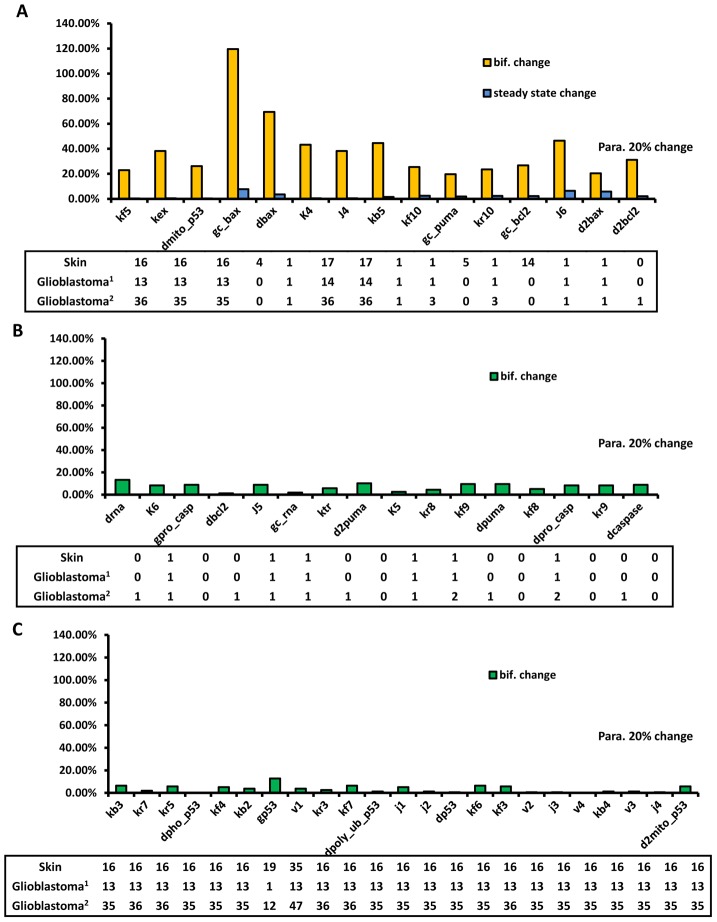
The correspondence between parameter sensitivities and the cancer gene mutation spectrum. (A) The correspondence between parameters linked to sensitivity of the bifurcation point (yellow bar) or caspase3 (blue bar) and high-frequency mutation genes. (B) The correspondence between insensitive parameters and low-frequency mutation genes. (C) The inconsistency between parameter sensitivity and gene mutation frequency. The numbers in the frame indicate the number of occurrences in the mutation spectrum of the gene that relates to the corresponding parameters.

As shown in Figure 4A, the critical points of bifurcation are largely affected by 15 parameters (yellow in Figure 4A), which we then selected to compare with the skin cancer and glioblastoma multiforme gene mutation spectrum, as shown in Figure 5A. Changes in the bifurcation point (yellow bar) and in the steady-state concentration of caspase3 (blue bar) are displayed alongside mutation frequencies in skin cancer and glioblastoma multiforme. Almost all influential parameters correspond to mutation hot spots in skin cancer and glioblastoma multiforme. This result supports our hypothesis that bifurcation points are sensitive to parameters corresponding to mutations that are most likely oncogenic. Here, we note that the definition of “mutation hot spot” is not quantitative; we will discuss this issue in the last section of the paper.

For the sake of comparison, we also selected the parameters to which the bifurcation point was insensitive; the result is presented in Figures 5B and 5C. We found that a part of the insensitive parameters correspond to very small number of mutation hot spots (Figure 5B), but the other part correspond to a large number of mutation hot spots (Figure 5C). Several factors may contribute to the inconsistency. It is well established that alteration of a single gene may not be oncogenic in itself [40]—in most cases, multiple hits are necessary [41]. We therefore suggest a synergistic effect for these parameters (Figure 5C), where two or more parameter changes, which are non-influential in isolation, may induce sensitivity in the bifurcation point when they co-occur. Indeed, we found that by decreasing kf3 (association rate of p53 and MDM2) and increasing kr3 (dissociation rate of p53/MDM2 complex) at the same time by 1.2-fold, the bifurcation point changes by about 22%. However, when changing these two parameters in isolation, the bifurcation point only changes by 5% and 2%. This may partially explain the observed inconsistency. Furthermore, one gene mutation may affect several parameters in our model, and one model parameter may involve several genes. In our analysis, the one-to-one mapping between the model parameters and the genes involved in the network is certainly an oversimplification. Fully understanding this behaviour requires detailed knowledge of the effect of mutations on the parameters, which is not available except in a few special cases.

**Figure 6:**
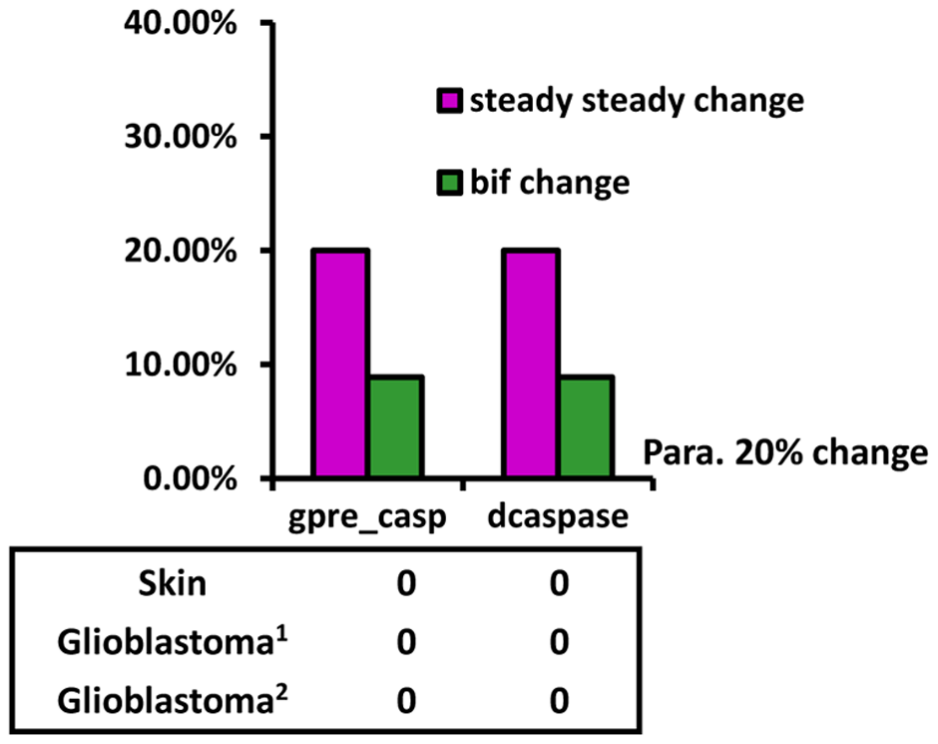
Comparison of parameters linked to sensitivity of caspase3 levels and gene mutations. The numbers in the frame indicate the number of occurrences in the mutation spectrum of the gene relating to the corresponding parameters. Magenta bar, change in the steady-state concentration of caspase3; Green bar, change in the critical point of bifurcation. gpre-casp, the basal generation rate of pre-caspase; dcaspase, the degradation rate of caspase3.

Similarly, we investigated the effect of different parameters on the steady state concentration of caspase3 as a measure of oncogenesis. We identified two parameters (magenta in Figure 4B), which led to the largest changes in the steady-state concentration of caspase3. We also calculated changes in the critical point when changing the parameters (Figure 6). According to the results in Figures 5A and 6, and compared with the results of bifurcation points, the relationship between the protein steady-state concentration and the cancer mutation spectrum is very weak.

## Discussion

Our work is based on the hypothesis that key regulators in physiological networks and oncogenesis are closely correlated and that the critical point of bifurcation is a good measure of network functionality. Therefore, mutations that cause variations in parameters that affect the bifurcation point are more likely to be oncogenic. In our apoptosis model, the location of the saddle-node bifurcation point reflects the DNA damage threshold where apoptosis is activated; when this threshold is exceeded, the system will switch from the low to the high state, which is accompanied by a rapid increase in caspase3 levels. A mutation may increase the apoptotic threshold, thereby allowing cells to evade apoptosis even at high levels of DNA damage, which may facilitate oncogenesis—a hypothesis that was confirmed by our analysis.

Distinguishing driver- from passenger-mutations is a central challenge in cancer research [42]–[44], and recently a network-based approach to identify cancer driver mutations has been proposed [6], [7]. Similarly, our strategy may be applied to identify driver mutations by identifying parameters with the greatest impact on the bifurcation point.

Several issues need to be addressed in this analysis: First, what is the impact of the Hopf bifurcation of nuclear p53 on oncogenesis? Although a number of studies have investigated the oscillatory behaviour of p53 in response to stress [25], [45]–[47], the functional role of these oscillations in DNA damage response remains unclear. We also conducted parameter sensitivity analysis of the Hopf bifurcation of nuclear p53 as a function of the level of DNA damage [12], and found a strong correlation between the spectrum of parameter sensitivities and the oncogenic mutation spectrum (see Figure S1). This may indicate that nuclear p53 oscillation plays a crucial role in protecting the cell against malignant transformation [11].

Second, as previously stated, our model parameters do not have one-to-one correspondence with gene mutations: changing one parameter may correspond to mutations in different genes or different types of mutations in the same gene. For example, the association and dissociation constants of two proteins may relate to mutations in either of the associated genes; an increase in a given protein may be caused by an increase in gene copy number, or by an increase in the catalytic efficiency of the relevant transcription factor (corresponding to the mutation). Moreover, different mutations in a single gene may correspond to different parameters: a gene mutation may change the functionality of the protein, reduce the binding capacity of the protein with another protein, or alter its phosphorylation efficiency. As molecular biology advances, information regarding the function of different mutations in regulatory networks will become more quantitative, which will allow for more precise analysis using our model.

The third concern relates to the definition of an oncogenic mutation hot spot. Different genes may be involved in different regulatory pathways, and have very large differences in mutation frequency. For example, p53 is involved in several regulatory pathways and has hundreds of known mutations, while Puma (*BBC3*) is involved in the apoptosis pathway, with only four or five known mutations. A method for normalising mutation frequency is necessary to allow quantitative analysis using our model. However, due to the lack of detailed information on the impact of each mutation on different regulatory pathways and on the model parameters, our analysis can only be qualitative. In this work, we used arbitrary thresholds to define a “mutation hot spot”, that were determined by our knowledge of the specific gene. As such, for genes involved in several regulatory pathways and with a high mutation frequency (like p53), we set a high threshold value (>10) for mutation hot spots. For genes involved in only one or two pathways, and which have very low mutation frequencies (like Puma), we believe that in spite of the low mutation frequency, they are still mutation hot spots. Because of the lack of detailed information on the impact of each mutation on the model parameters, our analysis can only achieve qualitative conclusions.

**Figure 7:**
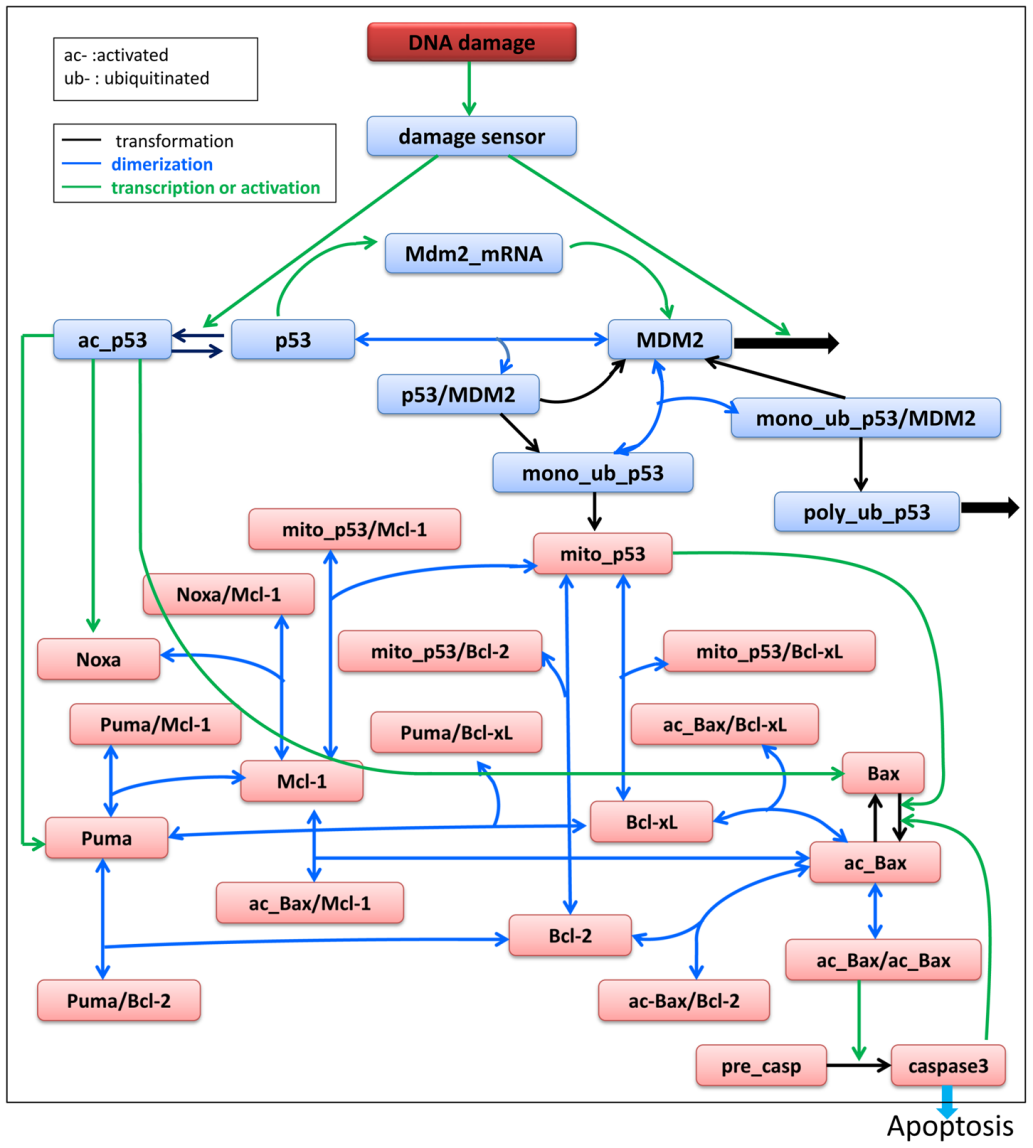
Extended DNA damage-induced apoptotic pathway. Blue lines indicate dimerization; black lines indicate transformation; green lines indicate transcription or activation. “mono-ub”, mono-ubiquitinated; “poly-ub”, poly-ubiquitinated; “mito-”, mitochondrial; “/” stands for complex.

**Figure 8:**
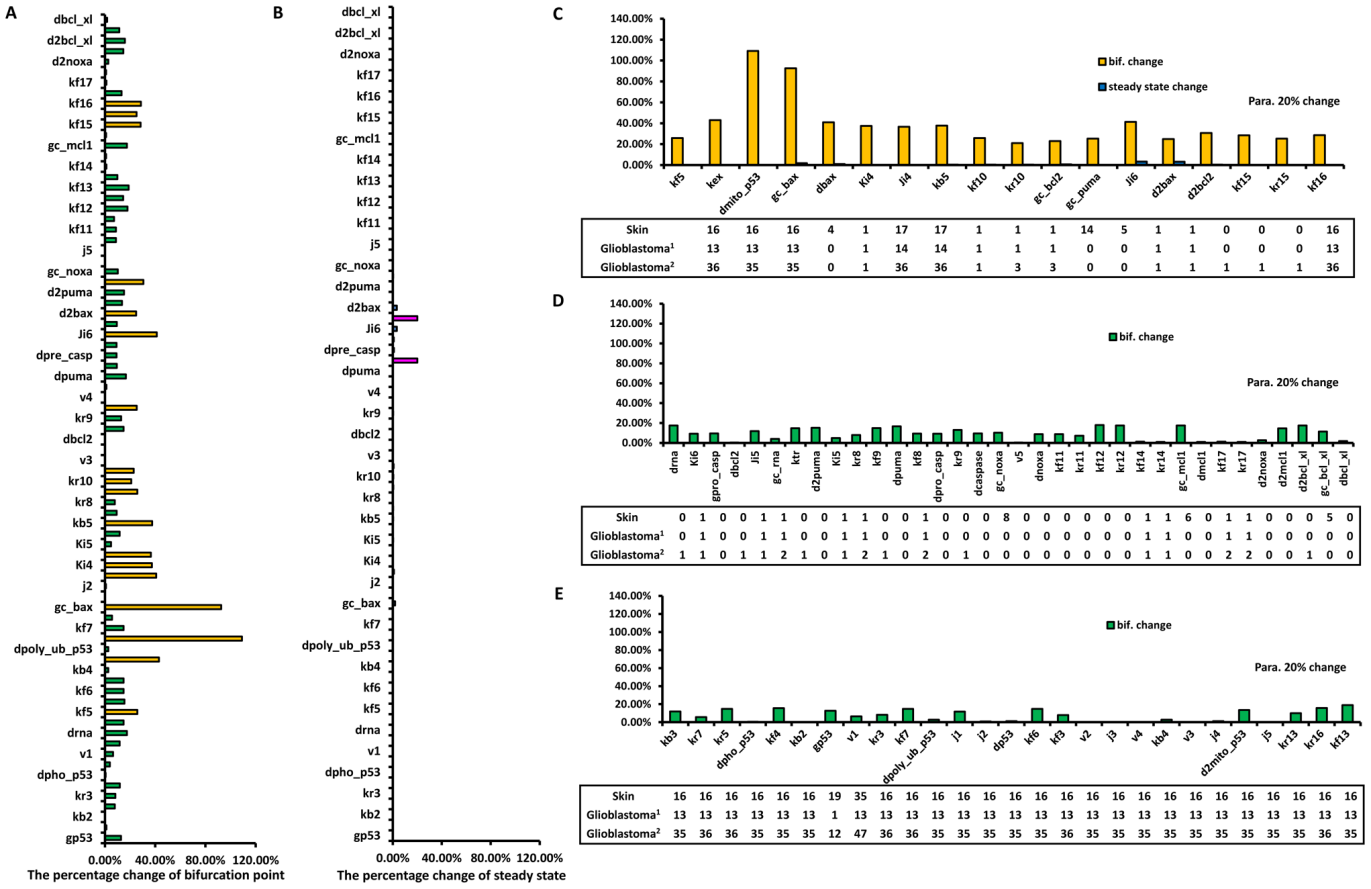
Parameter sensitivity analysis and its correspondence with mutations in the extended pathway. (A) The change in the saddle-node bifurcation point in response to a 20% increase or decrease of each parameter of the extended apoptosis pathway. Yellow and green bars indicate parameters that cause a large or small percentage change in the bifurcation points, respectively. (B) The change in the steady-state concentration of caspase-3 in response to a 20% increase or decrease in each parameter of the extended apoptosis pathway. Magenta and blue bars indicate parameters that cause a large or small change in the steady-state concentration of caspase3, respectively. (C) The correspondence between parameters linked to sensitivity of the bifurcation point (yellow bar) or caspase3 (blue bar) and high-frequency mutation genes. (D) The correspondence between insensitive parameters and low-frequency mutation genes. (E) The inconsistency between parameter sensitivity and gene mutation frequency. The numbers in the frame indicate the number of occurrences in the mutation spectrum of the gene that relates to the corresponding parameters.

The fourth issue relates to the simplicity of our model network: although the apoptosis pathway involves both the extrinsic and intrinsic pathways [48], we used a simplified, qualitative network model to conduct our research. To prove the validity of our model, we extended the current apoptosis pathway and repeated our analysis. Compared with the original model, the extended model consisted of 10 additional nodes including Noxa, Mcl-1, Bcl-xL and the complexes that they formed. Puma, Noxa, Bcl-2, Mcl-1 and Bcl-xL are all proteins of the Bcl-2 family. Like Puma, Noxa is a pro-apoptotic protein, which is regulated by nuclear p53 at the transcriptional level. Bcl-2, Mcl-1 and Bcl-xL are pro-survival proteins that inhibit cell apoptosis [48]. Puma binds Bcl-2, Bcl-xL and Mcl-1, whereas Noxa binds only Mcl-1 [49]. The corresponding extended regulatory network is shown in Figure 7. The details of the extended model are presented in the Supporting Information (Text S1, Figure S2and Figure S3). The results of the parameter sensitivity analysis and the correspondence between parameter sensitivity and mutations are shown in Figure 8. Notably, sensitive parameters in the extended pathway are very similar to those of the simplified pathway, with the 15 most influential parameters shared between the simplified (Figure 4A) and the extended pathways (Figure 8A). However, in the extended pathway, we found three additional sensitive parameters. Similar to the results of the simplified model (Figure 5A), we found that all sensitive parameters in the extended pathway correspond to mutation hot spots found in skin cancer and glioblastoma multiforme (Figure 8C). The analysis of the extended version of the network produces almost the same results as the simplified network, which supports the applicability and validity of our method.

Finally, in our analysis, the change of each mutated property (parameter in ODE) was counted only once within our model, despite the general consensus that more than one mutation is needed for oncogenesis to occur [50]. Our primary goal is to study the role of mutated genes in cancer-related biological functions. In future, we will analyse the role of multiple mutations on network functionality.

## Materials and Methods

### Equations for the DNA damage-induced apoptotic pathway

We compiled a set of ordinary differential equations (ODEs) (Text S1.) to model the apoptotic pathway in response to DNA damage. Model parameters were chosen based on the literature and biochemical constraints [51].

### Gene mutation database of cancer

The skin cancer mutated genes database was obtained from COSMIC (http://www.sanger.ac.uk/genetics/CGP/cosmic), glioblastoma multiforme mutated genes from the “CAN-genes” by Parsons et al. [4], and TCGA [5]. The “CAN-genes” by Parsons et al. [4] included genes frequently mutated in 22 glioblastoma multiforme samples. The TCGA project has catalogued somatic mutations and recurrent copy number alterations in 91 glioblastoma multiforme cases [5]. The basis and details of the correspondence of three forms of gene mutations and specific parameters in ordinary differential equations (ODEs) in our model are given in Text S1.

## Supporting Information

Figure S1 **Parameter sensitivity analysis and its correspondence with mutations.** (A) The percentage change in the Hopf bifurcation point in response to 20% increase or decrease in each parameter. (B) The correspondence between sensitive parameters and high-frequency mutation genes.
(TIF)

Figure S2 **Time evolution diagram of the level of total nuclear p53 and caspase3.** Blue and black lines represent p53 concentrations at low- and high-level DNA damage, respectively; Green and red lines represent caspase3 concentrations at low- and high-level DNA damage, respectively.
(TIF)

Figure S3 **Bifurcation diagram for caspase3 using DNA damage as the control parameter.**
(TIF)

Figure S4 **Comparison of parameters linked to sensitivity of caspase3 levels and gene mutations.**
(TIF)

Table S1 **The specific correspondence between each parameters and its gene mutations.** amp, amplification; mut, mutation; del, deletion.
(DOCX)

Table S2 **Parameter sensitivity analysis.** The effect of a 1.2-fold increase or decrease of each of the 54 parameters on the percentage change in the critical point of bifurcation.
(DOCX)

Table S3 **Parameter sensitivity analysis.** The effect of a 1.2-fold increase or decrease of each of the 54 parameters on the percentage change in the critical point of bifurcation.
(DOCX)

Text S1 **Detailed description of the model and parameters used.**
(DOCX)
