# Supplementary figures and images for: Correlation between Oncogenic Mutations and Parameter Sensitivity of the Apoptosis Pathway Model
Source: PLoS Comput Biol. 2014 Jan 23;10(1):e1003451. doi: 10.1371/journal.pcbi.1003451 (PMC3900373; doi:10.1371/journal.pcbi.1003451)

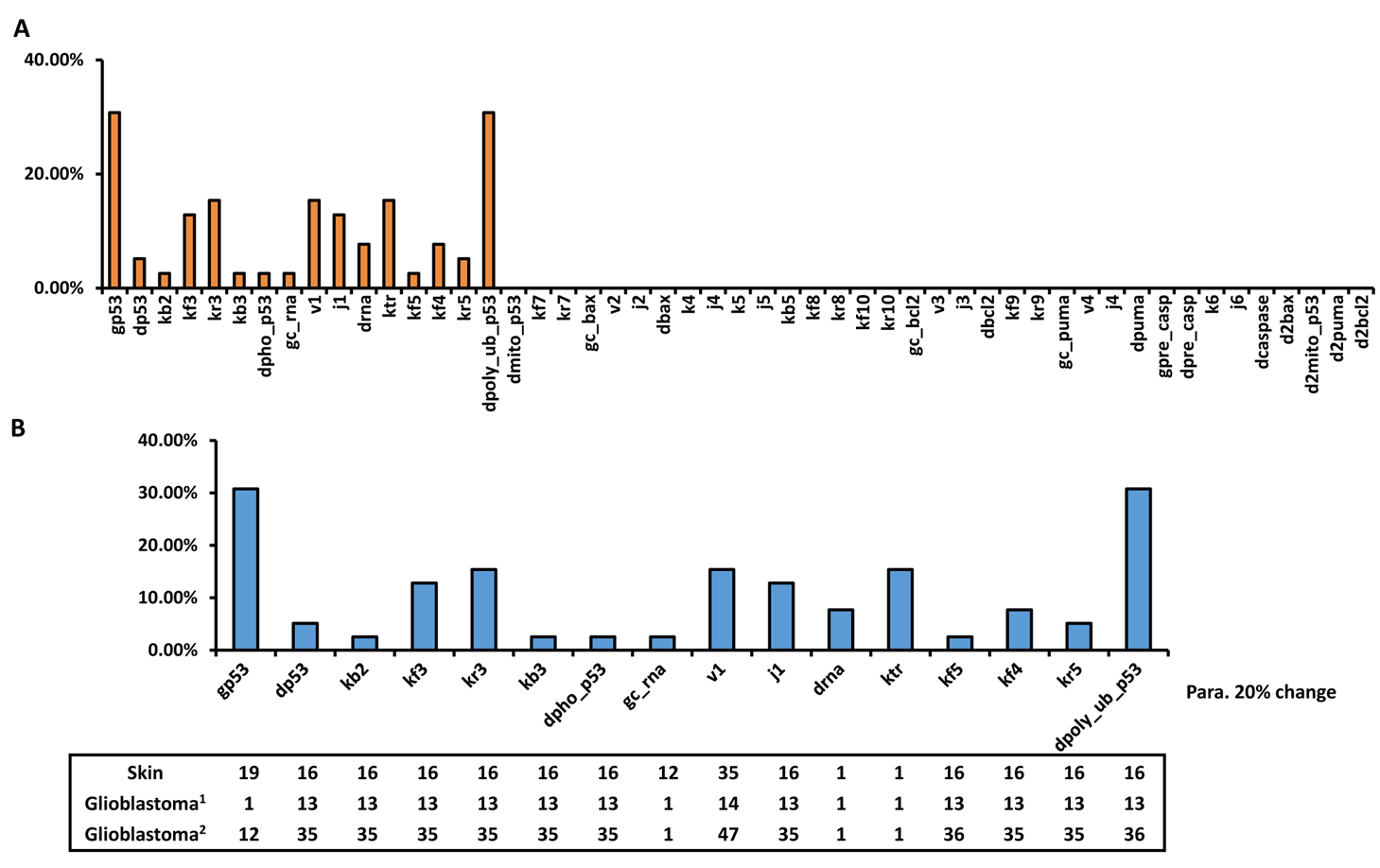

Supplement: Figure S1 — Parameter sensitivity analysis and its correspondence with mutations. (A) The percentage change in the Hopf bifurcation point in response to 20% increase or decrease in each parameter. (B) The correspondence between sensitive parameters and high-frequency mutation genes. (TIF) [file pcbi.1003451.s001.tif]

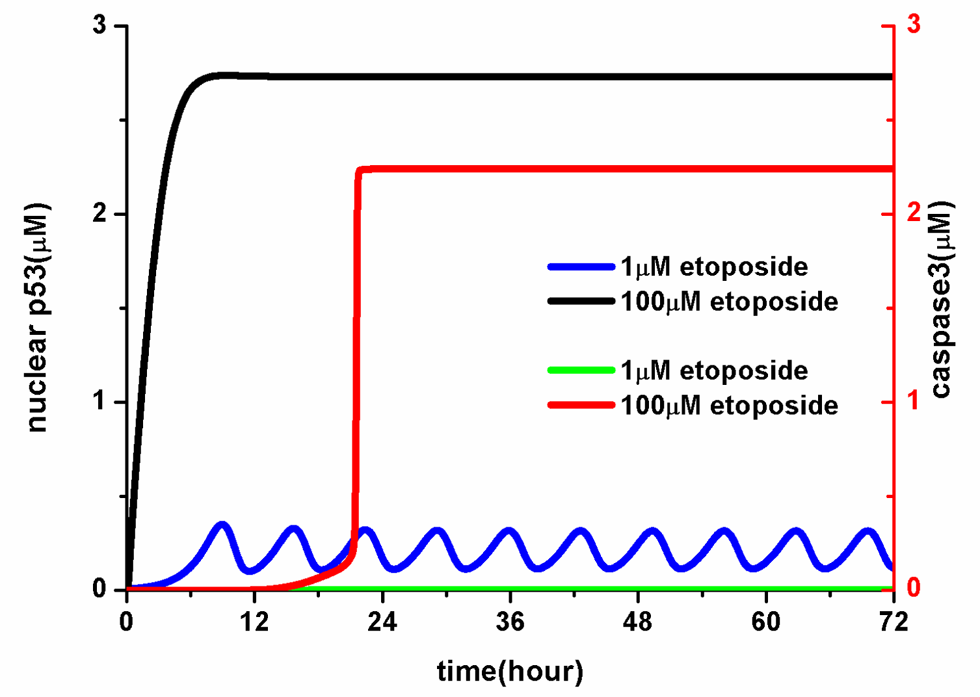

Supplement: Figure S2 — Time evolution diagram of the level of total nuclear p53 and caspase3. Blue and black lines represent p53 concentrations at low- and high-level DNA damage, respectively; Green and red lines represent caspase3 concentrations at low- and high-level DNA damage, respectively. (TIF) [file pcbi.1003451.s002.tif]

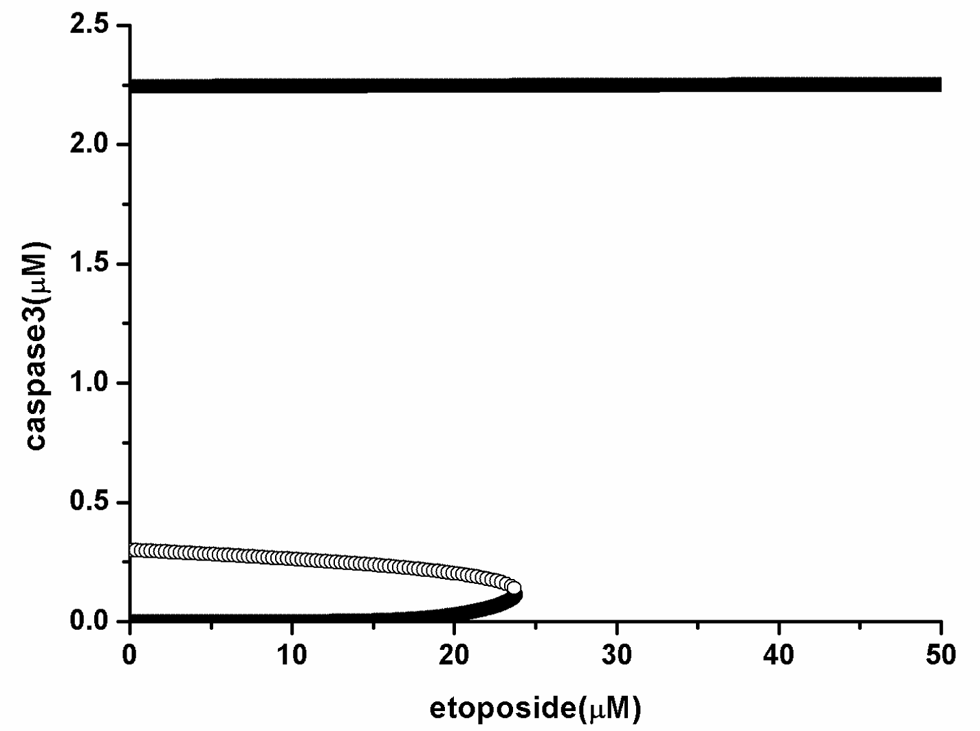

Supplement: Figure S3 — Bifurcation diagram for caspase3 using DNA damage as the control parameter. (TIF) [file pcbi.1003451.s003.tif]

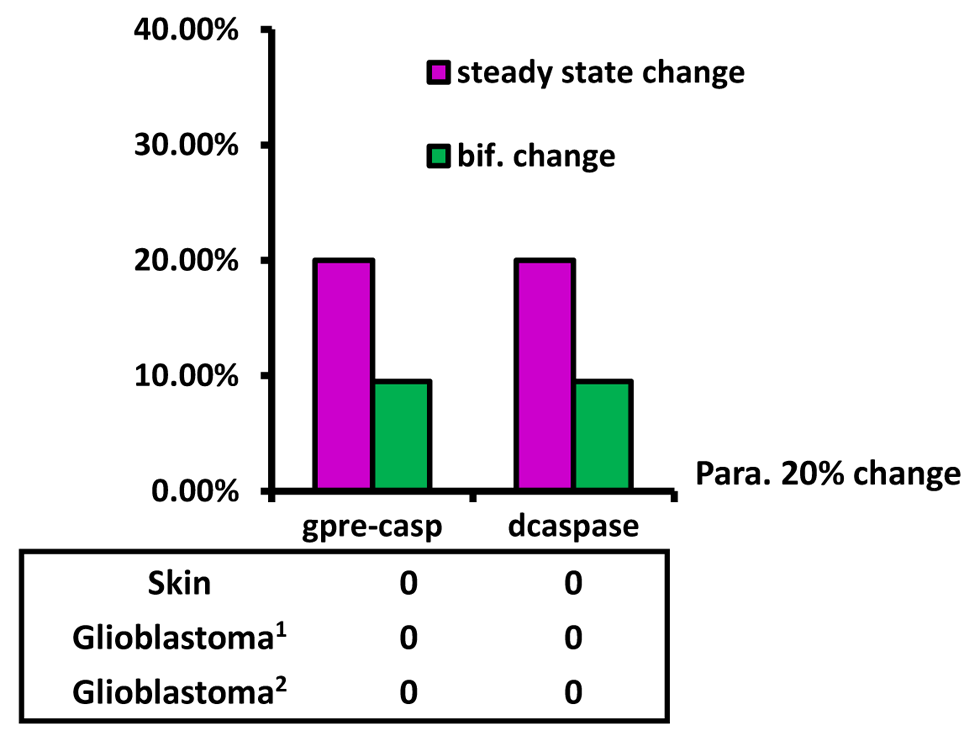

Supplement: Figure S4 — Comparison of parameters linked to sensitivity of caspase3 levels and gene mutations. (TIF) [file pcbi.1003451.s004.tif]
