# Supplementary material for: Correlation between Oncogenic Mutations and Parameter Sensitivity of the Apoptosis Pathway Model
Source: PLoS Comput Biol. 2014 Jan 23;10(1):e1003451. doi: 10.1371/journal.pcbi.1003451 (PMC3900373; doi:10.1371/journal.pcbi.1003451)
Supplement: Table S1 — The specific correspondence between each parameters and its gene mutations. amp, amplification; mut, mutation; del, deletion. (DOCX) [file pcbi.1003451.s005.docx]

Table S1.The specific corresponding of parameters and gene mutations. The meanings of abbreviation are: ‘amp’, amplification; ‘mut’, mutation; ‘del’, deletion.

| parameter | Corresponding gene mutation | |
| --- | --- | --- |
|  | 1.2-fold multiplication | 1.2-fold division |
| gp53 | amp:p53 | del:p53 |
| dp53 | mut:p53 | mut:p53 |
| kb2 | mut:p53 | mut:p53 |
| kf3 | mut:p53,mdm2 | mut:p53,mdm2 |
| kr3 | mut:p53,mdm2 | mut:p53,mdm2 |
| kb3 | mut:p53 | mut:p53 |
| dpho_p53 | mut:p53 | mut:p53 |
| gc_rna | amp:mdm2 | del:mdm2 |
| v1 | mut:p53 amp:mdm2 | mut:p53 del:mdm2 |
| j1 | mut:p53 | mut:p53 |
| drna | mut:mdm2 | mut:mdm2 |
| ktr | mut:mdm2 | mut:mdm2 |
| kf5 | mut:p53,mdm2 | mut:p53,mdm2 |
| kf4 | mut:p53 | mut:p53 |
| kf6 | mut:p53 | mut:p53 |
| kr5 | mut:p53,mdm2 | mut:p53,mdm2 |
| kb4 | mut:p53 | mut:p53 |
| kex | mut:p53 | mut:p53 |
| dpoly_ub_p53 | mut:p53 | mut:p53 |
| dmito_p53 | mut:p53 | mut:p53 |
| kf7 | mut:p53 bcl2 | mut:p53 bcl2 |
| kr7 | mut:p53 bcl2 | mut:p53 bcl2 |
| gc_bax | amp:bax | del:bax |
| v2 | mut:p53 amp:bax | mut:p53 del:bax |
| j2 | mut:p53 | mut:p53 |
| dbax | mut:bax | mut:bax |
| K4 | mut:p53 bax | mut:p53 bax |
| J4 | mut:p53 bax | mut:p53 bax |
| K5 | mut:casp bax | mut:casp bax |
| J5 | mut:casp bax | mut:casp bax |
| kb5 | mut:bax | mut:bax |
| kf8 | mut:bax bcl2 | mut:bax bcl2 |
| kr8 | mut:bax bcl2 | mut:bax bcl2 |
| kf10 | mut:bax | mut:bax |
| kr10 | mut:bax | mut:bax |
| gc_bcl2 | amp:bcl2 | del:bcl2 |
| v3 | mut:p53 amp:bcl2 | mut:p53 del:bcl2 |
| j3 | mut:p53 | mut:p53 |
| dbcl2 | mut:bcl2 | mut:bcl2 |
| kf9 | mut:puma bcl2 | mut:puma bcl2 |
| kr9 | mut:puma bcl2 | mut:puma bcl2 |
| gc_puma | amp:puma | del:puma |
| v4 | mut:p53 amp:puma | mut:p53 del:puma |
| j4 | mut:p53 | mut:p53 |
| dpuma | mut:puma | mut:puma |
| gpre_casp | amp:casp | del:casp |
| dpre_casp | mut:casp | mut:casp |
| K6 | mut:bax casp | mut:bax casp |
| J6 | mut:bax casp | mut:bax casp |
| dcaspase | mut:casp | mut:casp |
| d2bax | mut:bax | mut:bax |
| d2mito_p53; | mut:p53 | mut:p53 |
| d2puma; | mut:puma | mut:puma |
| d2bcl2; | mut:bcl2 | mut:bcl2 |
