# Supplementary material for: Correlation between Oncogenic Mutations and Parameter Sensitivity of the Apoptosis Pathway Model
Source: PLoS Comput Biol. 2014 Jan 23;10(1):e1003451. doi: 10.1371/journal.pcbi.1003451 (PMC3900373; doi:10.1371/journal.pcbi.1003451)
Supplement: Table S2 — Parameter sensitivity analysis. The effect of a 1.2-fold increase or decrease of each of the 54 parameters on the percentage change in the critical point of bifurcation. (DOCX) [file pcbi.1003451.s006.docx]

Table S2. Parameter sensitivity analysis. 1.2-fold multiplication and division change of a single parameter for all the 54 parameters and recording the percentage change of the critical point of bifurcation

| parameter | Location of Bif. point | 1.2-fold multiplication | | 1.2-fold division | |
| --- | --- | --- | --- | --- | --- |
|  |  | Location of Bif. point | change | Location of Bif. point | change |
| gp53 | 23.02 | 18.48 | -19.73% | 25.95 | 12.73% |
| dp53 | 23.02 | 22.87 | -0.64% | 23.17 | 0.64% |
| kb2 | 23.02 | 23.90 | 3.82% | 22.14 | -3.82% |
| kf3 | 23.02 | 21.41 | -7.00% | 24.34 | 5.73% |
| kr3 | 23.02 | 23.61 | 2.55% | 22.29 | -3.18% |
| kb3 | 23.02 | 25.07 | 8.91% | 20.53 | -10.82% |
| dpho_p53 | 23.02 | 23.02 | 0.00% | 23.02 | 0.00% |
| gc_rna | 23.02 | 23.46 | 1.91% | 22.73 | -1.27% |
| v1 | 23.02 | 23.90 | 3.82% | 21.70 | -5.73% |
| j1 | 23.02 | 20.38 | -11.45% | 25.66 | 11.45% |
| drna | 23.02 | 20.53 | -10.82% | 26.10 | 13.36% |
| ktr | 23.02 | 24.34 | 5.73% | 21.12 | -8.27% |
| kf5 | 23.02 | 28.29 | 22.91% | 17.75 | -22.91% |
| kf4 | 23.02 | 21.56 | -6.36% | 24.19 | 5.09% |
| kf6 | 23.02 | 24.49 | 6.36% | 21.41 | -7.00% |
| kr5 | 23.02 | 21.56 | -6.36% | 24.34 | 5.73% |
| kb4 | 23.02 | 22.73 | -1.27% | 23.31 | 1.27% |
| kex | 23.02 | 14.96 | -35.00% | 31.81 | 38.18% |
| dpoly_ub_ p53 | 23.02 | 23.31 | 1.27% | 22.73 | -1.27% |
| dmito_p53 | 23.02 | 29.03 | 26.09% | 17.31 | -24.82% |
| kf7 | 23.02 | 24.49 | 6.36% | 21.70 | -5.73% |
| kr7 | 23.02 | 22.58 | -1.91% | 23.46 | 1.91% |
| gc_bax | 23.02 | 8.96 | -61.09% | 50.56 | 119.63% |
| v2 | 23.02 | 22.87 | -0.64% | 23.17 | 0.64% |
| j2 | 23.02 | 23.31 | 1.27% | 22.73 | -1.27% |
| dbax | 23.02 | 38.99 | 69.36% | 11.89 | -48.36% |
| K4 | 23.02 | 14.38 | -37.54% | 32.98 | 43.27% |
| J4 | 23.02 | 31.81 | 38.18% | 14.96 | -35.00% |
| K5 | 23.02 | 22.58 | -1.91% | 23.61 | 2.55% |
| J5 | 23.02 | 25.07 | 8.91% | 20.97 | -8.91% |
| kb5 | 23.02 | 33.27 | 44.54% | 14.23 | -38.18% |
| kf8 | 23.02 | 24.19 | 5.09% | 22.00 | -4.45% |
| kr8 | 23.02 | 22.14 | -3.82% | 24.05 | 4.45% |
| kf10 | 23.02 | 17.16 | -25.45% | 28.88 | 25.45% |
| kr10 | 23.02 | 28.44 | 23.54% | 17.60 | -23.54% |
| gc_bcl2 | 23.02 | 29.17 | 26.73% | 18.77 | -18.45% |
| v3 | 23.02 | 23.31 | 1.27% | 22.87 | -0.64% |
| j3 | 23.02 | 23.17 | 0.64% | 22.87 | -0.64% |
| dbcl2 | 23.02 | 22.73 | -1.27% | 23.31 | 1.27% |
| kf9 | 23.02 | 20.82 | -9.54% | 25.22 | 9.54% |
| kr9 | 23.02 | 24.92 | 8.27% | 21.26 | -7.64% |
| gc_puma | 23.02 | 19.07 | -17.18% | 27.56 | 19.73% |
| v4 | 23.02 | 23.02 | 0.00% | 23.02 | 0.00% |
| j4 | 23.02 | 23.17 | 0.64% | 23.02 | 0.00% |
| dpuma | 23.02 | 25.22 | 9.54% | 20.82 | -9.54% |
| gpre_casp | 23.02 | 20.97 | -8.91% | 25.07 | 8.91% |
| dpre_casp | 23.02 | 24.92 | 8.27% | 21.12 | -8.27% |
| K6 | 23.02 | 21.12 | -8.27% | 24.92 | 8.27% |
| J6 | 23.02 | 33.71 | 46.45% | 14.82 | -35.63% |
| dcaspase | 23.02 | 25.07 | 8.91% | 20.97 | -8.91% |
| d2bax | 23.02 | 27.71 | 20.36% | 19.21 | -16.54% |
| d2mito_p53 | 23.02 | 24.34 | 5.73% | 21.85 | -5.09% |
| d2puma; | 23.02 | 25.36 | 10.18% | 21.26 | -7.64% |
| d2bcl2; | 23.02 | 18.04 | -21.64% | 30.20 | 31.18% |
