# Supplementary material for: Correlation between Oncogenic Mutations and Parameter Sensitivity of the Apoptosis Pathway Model
Source: PLoS Comput Biol. 2014 Jan 23;10(1):e1003451. doi: 10.1371/journal.pcbi.1003451 (PMC3900373; doi:10.1371/journal.pcbi.1003451)
Supplement: Table S3 — Parameter sensitivity analysis. The effect of a 1.2-fold increase or decrease of each of the 54 parameters on the percentage change in the critical point of bifurcation. (DOCX) [file pcbi.1003451.s007.docx]

Table S3. Parameter sensitivity analysis. 1.2-fold multiplication and division change of a single parameter for all the 54 parameters and recording the percentage change of the steady-state concentrations of caspase3

| parameter | steady-state concentrations | 1.2-fold multiplication | | 1.2-fold division | |
| --- | --- | --- | --- | --- | --- |
|  |  | steady-state concentrations | change | steady-state concentrations | change |
| gp53 | 2.05 | 2.06 | 0.39% | 2.04 | -0.37% |
| dp53 | 2.05 | 2.05 | -0.03% | 2.05 | 0.02% |
| kb2 | 2.05 | 2.05 | 0.06% | 2.05 | -0.07% |
| kf3 | 2.05 | 2.05 | 0.13% | 2.04 | -0.13% |
| kr3 | 2.05 | 2.05 | -0.09% | 2.05 | 0.08% |
| kb3 | 2.05 | 2.05 | -0.11% | 2.05 | 0.11% |
| dpho_p53 | 2.05 | 2.05 | -0.07% | 2.05 | 0.06% |
| gc_rna | 2.05 | 2.05 | -0.02% | 2.05 | 0.02% |
| v1 | 2.05 | 2.04 | -0.16% | 2.05 | 0.16% |
| j1 | 2.05 | 2.05 | 0.05% | 2.05 | -0.03% |
| drna | 2.05 | 2.05 | 0.18% | 2.04 | -0.17% |
| ktr | 2.05 | 2.04 | -0.17% | 2.05 | 0.18% |
| kf5 | 2.05 | 2.04 | -0.31% | 2.05 | 0.31% |
| kf4 | 2.05 | 2.05 | 0.08% | 2.05 | -0.09% |
| kf6 | 2.05 | 2.05 | -0.08% | 2.05 | 0.10% |
| kr5 | 2.05 | 2.05 | 0.10% | 2.05 | -0.08% |
| kb4 | 2.05 | 2.05 | 0.02% | 2.05 | -0.01% |
| kex | 2.05 | 2.06 | 0.42% | 2.04 | -0.41% |
| dpoly_ub_p53 | 2.05 | 2.05 | -0.01% | 2.05 | 0.02% |
| dmito_p53 | 2.05 | 2.04 | -0.34% | 2.05 | 0.33% |
| kf7 | 2.05 | 2.05 | -0.06% | 2.05 | 0.05% |
| kr7 | 2.05 | 2.05 | 0.02% | 2.05 | -0.02% |
| gc_bax | 2.05 | 2.21 | 7.74% | 1.92 | -6.34% |
| v2 | 2.05 | 2.06 | 0.60% | 2.04 | -0.53% |
| j2 | 2.05 | 2.05 | 0.00% | 2.05 | 0.00% |
| dbax | 2.05 | 1.93 | -5.67% | 2.12 | 3.58% |
| K4 | 2.05 | 2.06 | 0.47% | 2.04 | -0.44% |
| J4 | 2.05 | 2.04 | -0.40% | 2.06 | 0.41% |
| K5 | 2.05 | 2.07 | 1.29% | 2.02 | -1.55% |
| J5 | 2.05 | 2.05 | -0.03% | 2.05 | 0.01% |
| kb5 | 2.05 | 2.01 | -2.05% | 2.08 | 1.53% |
| kf8 | 2.05 | 2.02 | -1.53% | 2.07 | 1.25% |
| kr8 | 2.05 | 2.07 | 1.09% | 2.02 | -1.27% |
| kf10 | 2.05 | 2.10 | 2.51% | 1.98 | -3.27% |
| kr10 | 2.05 | 1.98 | -3.12% | 2.10 | 2.40% |
| gc_bcl2 | 2.05 | 1.97 | -3.79% | 2.09 | 2.21% |
| v3 | 2.05 | 2.05 | 0.00% | 2.05 | 0.00% |
| j3 | 2.05 | 2.05 | 0.00% | 2.05 | 0.00% |
| dbcl2 | 2.05 | 2.05 | 0.14% | 2.04 | -0.12% |
| kf9 | 2.05 | 2.07 | 1.19% | 2.02 | -1.42% |
| kr9 | 2.05 | 2.02 | -1.21% | 2.07 | 1.02% |
| gc_puma | 2.05 | 2.09 | 1.84% | 1.99 | -2.57% |
| v4 | 2.05 | 2.05 | 0.06% | 2.05 | -0.05% |
| j4 | 2.05 | 2.05 | 0.00% | 2.05 | 0.00% |
| dpuma | 2.05 | 2.02 | -1.42% | 2.07 | 1.19% |
| gpre_casp | 2.05 | 2.46 | 20.02% | 1.71 | -16.69% |
| dpre_casp | 2.05 | 2.00 | -2.53% | 2.09 | 2.21% |
| K6 | 2.05 | 2.09 | 2.21% | 2.00 | -2.53% |
| J6 | 2.05 | 1.82 | -11.14% | 2.18 | 6.42% |
| dcaspase | 2.05 | 1.71 | -16.69% | 2.46 | 20.02% |
| d2bax | 2.05 | 1.85 | -9.75% | 2.17 | 5.77% |
| d2mito_p53 | 2.05 | 2.05 | -0.07% | 2.05 | 0.06% |
| d2puma; | 2.05 | 2.02 | -1.23% | 2.06 | 0.86% |
| d2bcl2; | 2.05 | 2.09 | 2.17% | 1.97 | -3.57% |
