# Supplementary material for: Correlation between Oncogenic Mutations and Parameter Sensitivity of the Apoptosis Pathway Model
Source: PLoS Comput Biol. 2014 Jan 23;10(1):e1003451. doi: 10.1371/journal.pcbi.1003451 (PMC3900373; doi:10.1371/journal.pcbi.1003451)
Supplement: Text S1 — Detailed description of the model and parameters used. (DOCX) [file pcbi.1003451.s008.docx]

## Supporting Information

**TextS1**

1. **Models and ODEs**

1.1 Rules in translation of regulatory network to ODEs

Our model is formulated as a set of coupled Ordinary Differential Equations (ODEs):


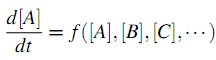


Where A, B, C … represent the reactants, such as proteins, enzymes, mRNA et al., their dynamics are governed by the function f. The derivation of these functions is according to the following rules:

1. Binding reactions,
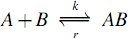


The dynamics is following the law of mass action:


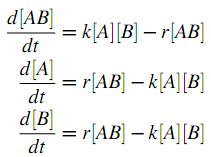


1. Enzyme catalysis,
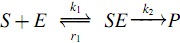


The dynamics of this reaction is following the Michaelis-Menten kinetics:


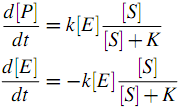


1. The promotion and inhibition of gene transcription .Transcriptional regulation is using Hill functions:


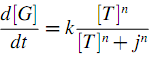


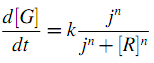


1.2. Apoptosis pathway in response to DNA damage

The Esq. (1)-(19) are the ODEs that translated according to the regulatory network of Fig. 1. They describe the dynamics of the apoptosis pathway in response to DNA damage. Damage is a control parameter here. This approach is a modification and application of the p53 DNA damage response network analysis described by Li, Z., et al. [[1](#_ENREF_1)]. Based on the different post-translational modifications of p53, active p53 can be distinguished into inducing different events [[2](#_ENREF_2)]. For simplicity we do not include the cell cycle arrest here, in our model, ac-p53 refers to phosphorylated p53 inducing apoptosis. Comparing with previous apoptosis model that pay more attention to the downstream events of mitochondria outer membrane permeability [[3](#_ENREF_3),[4](#_ENREF_4)], this model is a relatively simple one, with focus on the interactions of Bcl2 family proteins, which are directly influenced by p53. As several pieces of experimental work, it appears that p53 regulate apoptosis by modulating its direct pro-death activity in the cytoplasm, not through altering its transcriptional activity [[5](#_ENREF_5)].

1. ${\text{d[}\text{p}\text{53]}}/\text{dt}\text{=}\text{g}_{\text{p}\text{53}}\text{-}\text{d}_{\text{p}\text{53}}\left[ \text{p}\text{53} \right]\text{-}\text{k}_{\text{a}\text{2}}\left[ \text{p}\text{53} \right]\text{+}\text{k}_{\text{b}\text{2}}\left[ \text{pho}\text{\_}\text{p}\text{53} \right]\text{-}\text{k}_{\text{f}\text{3}}\left[ \text{p}\text{53} \right]\left[ \text{Mdm}\text{2} \right]\text{+}$

$$\text{k}_{\text{r}\text{3}}\text{[}\text{p}\text{53/}\text{Mdm}\text{2]+}\text{k}_{\text{b}\text{3}}\text{[}\text{mono}\text{\_}\text{ub}\text{\_}\text{p}\text{53]}$$

$$(2){d[pho\_p53]}/\mathrm{dt}=k_{a2}\left[ p53 \right]-k_{b2}\left[ pho\_p53 \right]-d_{pho\_p53}\left[ pho\_p53 \right]$$

$$(3){d[\mathrm{RNA}]}/\mathrm{dt}=g_{c\_\mathrm{RNA}}+V_{1}\frac{{[p53]}^{4}}{{[p53]}^{4}+{j_{1}}^{4}}-d_{\mathrm{RNA}}[\mathrm{RNA}]$$

$$\text{(4)}{\text{d}\text{[}\text{Mdm}\text{2]}}/\text{dt}\text{=}\text{k}_{\text{tr}}\left[ \text{RNA} \right]\text{-}\text{d}_{\text{Mdm}\text{2}}\left[ \text{Mdm}\text{2} \right]\text{-}\text{k}_{\text{f}\text{3}}\left[ \text{p}\text{53} \right]\left[ \text{Mdm}\text{2} \right]\text{-}\text{k}_{\text{f}\text{5}}\left[ \text{mono}\text{\_}\text{ub}\text{\_}\text{p}\text{53} \right]\left[ \text{Mdm}\text{2} \right]$$

$$\text{ }\text{+(}\text{k}_{\text{r}\text{3}}\text{+}\text{k}_{\text{f}\text{4}}\text{)[}\text{p}\text{53/}\text{Mdm}\text{2]+(}\text{k}_{\text{r}\text{5}}\text{+}\text{k}_{\text{f}\text{6}}\text{)}\left[ \text{mono}\text{\_}\text{ub}\text{\_}\text{p}\text{53/}\text{Mdm}\text{2} \right]$$

$$(5){d[p53/\mathrm{Mdm}2]}/\mathrm{dt}=k_{f3}\left[ p53 \right]\left[ \mathrm{Mdm}2 \right]-(k_{r3}+k_{f4})[p53/\mathrm{Mdm}2]$$

(6)${\text{d [}\text{mono}\text{\_}\text{ub}\text{\_}\text{p}\text{53 ]}}/\text{dt}\text{=}\text{kf}\text{4 [}\text{p}\text{53/}\text{Mdm}\text{2]}\text{-kb}\text{3 [}\text{mono}\text{\_}\text{ub}\text{\_}\text{p}\text{53 ]}\text{-}$

$${\text{ }\text{ }\text{k}}_{\text{f}\text{5}}\left[ \text{mono}\text{\_}\text{ub}\text{\_}\text{p}\text{53} \right]\left[ \text{Mdm}\text{2} \right]\text{+}\text{k}_{\text{r}\text{5}}\left[ \text{mono}\text{\_}\text{ub}\text{\_}\text{p}\text{53/}\text{Mdm}\text{2} \right]\text{ }$$

$$\text{ }\text{+}{\text{ }\text{k}}_{\text{b}\text{4}}\text{[}\text{poly}\text{\_}\text{ub}\text{\_}\text{p}\text{53]}\text{-}\text{k}_{\text{ex}}\text{[}\text{mono}\text{\_}\text{ub}\text{\_}\text{p}\text{53]}$$

$$\left( \text{7} \right){\text{d}\left[ \text{mono}\text{\_}\text{ub}\text{\_}\text{p}\text{53/}\text{Mdm}\text{2} \right]}/\text{dt}\text{=}\text{k}_{\text{f}\text{5}}\left[ \text{mono}\text{\_}\text{ub}\text{\_}\text{p}\text{53} \right]\left[ \text{Mdm}\text{2} \right]\text{-}$$

$$\text{ }\text{(}\text{k}_{\text{r}\text{5}}\text{+}\text{k}_{\text{f}\text{6}}\text{)}\left[ \text{mono}\text{\_}\text{ub}\text{\_}\text{p}\text{53/}\text{Mdm}\text{2} \right]$$

$$\left( \text{8} \right){\text{d[}\text{poly}\text{\_}\text{ub}\text{\_}\text{p}\text{53]}}/\text{dt}\text{=}\text{k}_{\text{f}\text{6}}\left[ \text{mono}\text{\_}\text{ub}\text{\_}\text{p}\text{53/}\text{Mdm}\text{2} \right]\text{-}\text{(}\text{k}_{\text{b}\text{4}}\text{+}\text{d}_{\text{poly}\text{\_}\text{ub}\text{\_}\text{p}\text{53}}\text{)[}\text{poly}\text{\_}\text{ub}\text{\_}\text{p}\text{53]}$$

$$\left( 9 \right){d\left[ \mathrm{mito}\_p53 \right]}/\mathrm{dt}=k_{\mathrm{ex}}\left[ \mathrm{mono}\_\mathrm{ub}\_p53 \right]-d_{\mathrm{mit}o_{p53}}\left[ \mathrm{mito}\_p53 \right]-$$

$$\text{ k}_{\text{f}\text{7}}\left[ \text{Bcl}\text{2} \right]\left[ \text{mito}\text{\_}\text{p}\text{53} \right]\text{+}\text{k}_{\text{r}\text{7}}\text{[}\text{mito}\text{\_}\text{p}\text{53/}\text{Bcl}\text{2]+}\text{d'}_{\text{bcl}\text{2}}\text{[}\text{mito}\text{\_}\text{p}\text{53/}\text{Bcl}\text{2]}$$

$$\text{(10)}{\text{d}\text{[}\text{Bax}\text{]}}/\text{dt}\text{=}\text{g}_{\text{c}\text{\_}\text{Bax}}\text{+}\text{V}_{\text{2}}\frac{{\text{[}\text{pho}\text{\_}\text{p}\text{53]}}^{\text{4}}}{{\text{[}\text{pho}\text{\_}\text{p}\text{53]}}^{\text{4}}\text{+}{\text{j}_{\text{2}}}^{\text{4}}}\text{-}\text{d}_{\text{Bax}}\left[ \text{Bax} \right]\text{-}\text{(}\text{K}_{\text{4}}\frac{\text{[}\text{mito}\text{\_}\text{p}\text{53]}}{\text{[}\text{mito}\text{\_}\text{p}\text{53]+}\text{J}_{\text{4}}}$$

$$\text{ }\text{+}\text{K}_{\text{5}}\frac{{\text{[}\text{caspase}\text{]}}^{\text{4}}}{{\text{[}\text{caspase}\text{]}}^{\text{4}}\text{+}{\text{J}_{\text{5}}}^{\text{4}}}\text{)[}\text{Bax}\text{]+}\text{k}_{\text{b}\text{5}}\text{[}\text{ac}\text{\_}\text{Bax}\text{]}$$

$$\left( 11 \right){\text{d}\left[ \text{ac\_Bax} \right]}/\text{dt}\text{=}\left( \text{K}_{\text{4}}\frac{\left[ \text{mit}\text{o}_{\text{p}\text{53}} \right]}{\left[ \text{mit}\text{o}_{\text{p}\text{53}} \right]\text{+}\text{J}_{\text{4}}}\text{+}\text{K}_{\text{5}}\frac{\left[ \text{caspase} \right]^{\text{4}}}{\left[ \text{caspase} \right]^{\text{4}}\text{+}{\text{J}_{\text{5}}}^{\text{4}}} \right)\left[ \text{Bax} \right]\text{-}{\text{ }\text{ }\text{k}}_{\text{b}\text{5}}\left[ \text{ac\_Bax} \right]\text{-}$$

$${\text{ }\text{ }\text{d}}_{\text{bax}}\left[ \text{ac\_Bax} \right]\text{-}\text{k}_{\text{f}\text{8}}\left[ \text{ac\_Bax} \right]\left[ \text{Bcl}\text{2} \right]\text{+}\text{k}_{\text{r}\text{8}}\text{[}\text{ac}\text{\_}\text{Bax}\text{/}\text{ac\_Bax}\text{]}\text{-}\text{ 2}\text{k}_{\text{f}\text{10}}\left[ \text{ac}_{\text{Bax}} \right]^{\text{2}}$$

$$\text{ }\text{+2}\text{k}_{\text{r}\text{10}}\text{[}\text{ac}\text{\_}\text{Bax}\text{/}\text{ac\_Bax}\text{]+}\text{d'}_{\text{bcl}\text{2}}\text{[}\text{ac}\text{\_}\text{Bax}\text{/}\text{ac\_Bax}\text{]+ 2}\text{d'}_{\text{bax}}\text{[}\text{ac}\text{\_}\text{Bax}\text{/}\text{ac\_Bax}\text{]}$$

$$\text{(12)}{\text{d}\text{[}\text{Bcl}\text{2]}}/\text{dt}\text{=}\text{g}_{\text{c}\text{\_}\text{Bcl}\text{2}}\text{+}\text{V}_{\text{3}}\frac{{\text{j}_{\text{3}}}^{\text{4}}}{{{\text{[}\text{pho}\text{\_}\text{p}\text{53]}}^{\text{4}}\text{+}\text{j}_{\text{3}}}^{\text{4}}}\text{-}\text{d}_{\text{Bcl}\text{2}}\left[ \text{Bcl}\text{2} \right]\text{-}\text{k}_{\text{f}\text{8}}\left[ \text{Bcl}\text{2} \right]\text{[}\text{Bax}\text{] }$$

$$\text{ }\text{+}\text{k}_{\text{r}\text{8}}\text{[}\text{ac}\text{\_}\text{Bax}\text{/}\text{Bcl}\text{2]}\text{-}\text{k}_{\text{f}\text{7}}\text{[}\text{Bcl}\text{2][}\text{mito}\text{\_}\text{p}\text{53]+}\text{k}_{\text{r}\text{7}}\text{[}\text{mito}\text{\_}\text{p}\text{53/}\text{Bcl}\text{2]}\text{-}$$

$$\text{k}_{\text{f}\text{9}}\text{[}\text{Puma}\text{][}\text{Bcl}\text{2]+}\text{k}_{\text{r}\text{9}}\text{[}\text{Puma}\text{/}\text{Bcl}\text{2]+}\text{d'}_{\text{bax}}\text{[}\text{ac}\text{\_}\text{Bax}\text{/}\text{Bcl}\text{2]}$$

$$\text{ }\text{+}\text{d'}_{\text{mito}\text{\_}\text{p}\text{53}}\text{[}\text{mito}\text{\_}\text{p}\text{53/}\text{Bcl}\text{2]+}\text{d'}_{\text{puma}}\text{[}\text{Puma}\text{/}\text{Bcl}\text{2]}$$

$$\left( \text{13} \right){\text{d}\left[ \text{Puma} \right]}/\text{dt}\text{=}\text{g}_{\text{c}_{\text{Puma}}}\text{+}\text{V}_{\text{4}}\frac{\left[ \text{ph}\text{o}_{\text{p}\text{53}} \right]^{\text{4}}}{{\left[ \text{ph}\text{o}_{\text{p}\text{53}} \right]^{\text{4}}\text{+}\text{j}_{\text{4}}}^{\text{4}}}\text{-}\text{ }\text{d}_{\text{Puma}}\left[ \text{Puma} \right]\text{-}\text{k}_{\text{f}\text{9}}\left[ \text{Puma} \right]\left[ \text{Bcl}\text{2} \right]$$

$$\text{ }\text{+}\text{k}_{\text{r}\text{9}}\text{[}\text{Puma}\text{/}\text{Bcl}\text{2]+}\text{d'}_{\text{bcl}\text{2}}\text{[}\text{Puma}\text{/}\text{Bcl}\text{2]}$$

$(14){d[\mathrm{ac}\_\mathrm{Bax}/ac\_Bax]}/\mathrm{dt}=k_{f8}\left[ \mathrm{Bcl}2 \right]\left[ \mathrm{ac}\_\mathrm{Bax} \right]-k_{r8}[\mathrm{ac}\_\mathrm{Bax}/\mathrm{Bcl}2]-$(${d'}_{\mathrm{bax}}+ {d'}_{\mathrm{bcl}2})[\mathrm{ac}\_\mathrm{Bax}/\mathrm{Bcl}2]$

$${\left( 15 \right)\text{d}\text{[}\text{mito}\text{\_}\text{p}\text{53/}\text{Bcl}\text{2]}}/\text{dt}\text{=}\text{k}_{\text{f}\text{7}}\left[ \text{Bcl}\text{2} \right]\left[ \text{mito}\text{\_}\text{p}\text{53} \right]\text{-}\text{k}_{\text{r}\text{7}}\text{[}\text{mito}\text{\_}\text{p}\text{53/}\text{Bcl}\text{2]}\text{-}\text{ }$$

$$\text{ }\text{( }\text{d'}_{\text{mito}\text{\_}\text{p}\text{53}}\text{+ }\text{d'}_{\text{bcl}\text{2}}\text{)[}\text{mito}\text{\_}\text{p}\text{53/}\text{Bcl}\text{2]}$$

$$\left( 16 \right){\text{d}\text{[}\text{Puma}\text{/}\text{Bcl}\text{2]}}/\text{dt}\text{=}\text{k}_{\text{f}\text{9}}\left[ \text{Puma} \right]\left[ \text{Bcl}\text{2} \right]\text{-}\text{k}_{\text{r}\text{9}}\text{[}\text{Puma}\text{/}\text{Bcl}\text{2]}\text{-}\text{(}\text{d'}_{\text{puma}}\text{+}\text{ d'}_{\text{bcl}\text{2}}\text{)[}\text{Puma}\text{/}\text{Bcl}\text{2]}$$

$$\left( \text{17} \right){\text{d[}\text{ac}\text{\_}\text{Bax}\text{/}\text{ac}\text{\_}\text{Bax}\text{]}}/\text{dt}\text{=}\text{k}_{\text{f}\text{10}}\left[ \text{ac\_Bax} \right]^{\text{2}}\text{-}\text{k}_{\text{r}\text{10}}\text{[}\text{ac}\text{\_}\text{Bax}\text{/}\text{ac}\text{\_}\text{Bax}\text{]}\text{-}\text{ d'}_{\text{bax}}\text{[}\text{ac}\text{\_}\text{Bax}\text{/}\text{ac}\text{\_}\text{Bax}\text{]}$$

$$\left( \text{18} \right){\text{d[}\text{pre}\text{\_}\text{casp}\text{]}}/\text{dt}\text{=}\text{g}_{\text{pr}\text{e}_{\text{casp}}}\text{-}\text{ }\text{d}_{\text{pre}\text{\_}\text{casp}}\text{[}\text{pre}\text{\_}\text{casp}\text{]}$$

$$\text{ -}\text{K}_{\text{6}}\frac{{\text{[}\text{ac}\text{\_}\text{Bax}\text{/}\text{ac}\text{\_}\text{Bax}\text{]}}^{\text{4}}}{{\text{[}\text{ac}\text{\_}\text{Bax}\text{/}\text{ac}\text{\_}\text{Bax}\text{]}}^{\text{4}}\text{+}{\text{J}_{\text{6}}}^{\text{4}}}\text{[}\text{pre}\text{\_}\text{casp}\text{]}$$

$$(19){d[\mathrm{caspase}]}/\mathrm{dt}=K_{6}\frac{{[\mathrm{ac}\_\mathrm{Bax}/\mathrm{ac}\_\mathrm{Bax}]}^{4}}{{[\mathrm{ac}\_\mathrm{Bax}/\mathrm{ac}\_\mathrm{Bax}]}^{4}+{J_{6}}^{4}}\left[ \mathrm{pre}\_\mathrm{casp} \right]-d_{\mathrm{caspase}}[\mathrm{caspase}]$$

- 1. Extended apoptosis pathway in response to DNA damage

We added 10 nodes to our apoptosis pathway in response to DNA damage which are Noxa, Mcl-1, Bcl-xl and complexes that they formed. Puma, Noxa, Bcl-2, Mcl-1 and Bcl-xl are all the proteins of Bcl-2 family. Same as Puma, Noxa is a pro-apoptotic protein, of which nuclear p53 regulates transcription. Bcl-2, Mcl-1 and Bcl-xl are the pro-survival proteins that inhibit cell apoptosis [[6](#_ENREF_6)]. Puma binds Bcl-2, Bcl-xl and Mcl-1, whereas Noxa binds only Mcl-1 [[7](#_ENREF_7)]. The corresponding regulatory network is shown schematically in Fig. S2.

The Esq. (1)-(29) are the ODEs that translated according to the regulatory network of Fig. 8.

$$\text{(20)d[Noxa]}/\text{dt}\text{=}\text{g}_{\text{c}_{\text{Noxa}}}\text{+}\text{V}_{\text{5}}\frac{\left[ \text{ph}\text{o}_{\text{p53}} \right]^{\text{4}}}{{\left[ \text{ph}\text{o}_{\text{p53}} \right]^{\text{4}}\text{+}\text{j}_{\text{5}}}^{\text{4}}}\text{- }\text{d}_{\text{Noxa}}\left[ \text{Noxa} \right]\text{-}\text{k}_{\text{f11}}\left[ \text{Noxa} \right]\left[ \text{Mcl-1} \right]$$

$$\text{ }\text{+}\text{k}_{\text{r}\text{11}}\text{[}\text{Nox}\text{a/}\text{Mcl-1}\text{]+}\text{d'}_{\text{Mcl-1}}\text{[}\text{Nox}\text{a/}\text{Mcl-1}\text{]}$$

$$\text{(}\text{21}\text{)}{\text{d[}\text{Mcl-1}\text{]}}/\text{dt}\text{=}\text{g}_{\text{c\_}\text{Mcl-1}}\text{-}\text{d}_{\text{Mcl-1}}\left[ \text{Mcl-1} \right]\text{-}\text{k}_{\text{f}\text{11}}\left[ \text{Noxa} \right]\text{[}\text{Mcl-1}\text{] +}\text{k}_{\text{r}\text{11}}\text{[}\text{Noxa}\text{/}\text{Mcl-1}\text{]}$$

$$\text{ }\text{-} \text{k}_{\text{f}\text{12}}\text{[Puma][}\text{Mcl-1}\text{]+}\text{k}_{\text{r}\text{12}}\text{[Puma/}\text{Mcl-1}\text{]-}\text{k}_{\text{f}\text{13}}\text{[mito\_p53][}\text{Mcl-1}\text{]}$$

$$\text{ }\text{+}\text{k}_{\text{r}\text{13}}\text{[mito\_p53/}\text{Mcl-1}\text{]-}\text{k}_{\text{f}\text{14}}\left[ \text{ac\_Bax} \right]\text{[}\text{Mcl-1}\text{] +}\text{k}_{\text{r}\text{14}}\text{[ac\_Bax/}\text{Mcl-1}\text{]}$$

$$\text{+}\text{d'}_{\text{ac-}\text{bax}}\text{[ac\_Bax/}\text{Mcl-1}\text{]}\text{ }\text{+}\text{d'}_{\text{mito\_p53}}\text{[mito\_p53/}\text{Mcl-1}\text{]}$$

$$\text{ }\text{+}\text{d'}_{\text{puma}}\text{[Puma/}\text{Mcl-1}\text{]}\text{+}\text{d'}_{\text{Nox}\text{a}}\text{[}\text{Nox}\text{a/}\text{Mcl-1}\text{]}$$

$$\text{(}\text{22}\text{)}{\text{d[}\text{Bcl-xl}\text{]}}/\text{dt}\text{=}\text{g}_{\text{c\_}\text{Bcl-xl}}\text{-}\text{d}_{\text{Bcl-xl}}\left[ \text{Bcl-xl} \right]\text{-} \text{k}_{\text{f}\text{15}}\text{[Puma][}\text{Bcl-xl}\text{]+}\text{k}_{\text{r}\text{15}}\text{[Puma/}\text{Bcl-xl}\text{]}$$

$$\text{ }\text{-}\text{k}_{\text{f}\text{16}}\text{[mito\_p53][}\text{Bcl-xl}\text{] +}\text{k}_{\text{r}\text{16}}\text{[mito\_p53/}\text{Bcl-xl}\text{]-}\text{k}_{\text{f}\text{17}}\left[ \text{ac\_Bax} \right]\text{[}\text{Bcl-xl}\text{] }$$

$$\text{ }\text{+}\text{k}_{\text{r}\text{17}}\text{[ac\_Bax/}\text{Bcl-xl}\text{] +}\text{d'}_{\text{ac-}\text{bax}}\text{[ac\_Bax/}\text{Bcl-xl}\text{]+}\text{d'}_{\text{puma}}\text{[Puma/}\text{Bcl-xl}\text{]}$$

$\text{ }\text{+}\text{d'}_{\text{mito\_p53}}\text{[mito\_p53/}\text{Bcl-xl}\text{]}$

$$\text{(}\text{23}\text{)}{\text{d[}\text{Noxa/}\text{Mcl-1}\text{]}}/\text{dt}\text{=}\text{k}_{\text{f}\text{11}}\left[ \text{Noxa} \right]\text{[}\text{Mcl-1}\text{] }\text{–}\text{k}_{\text{r}\text{11}}\text{[}\text{Noxa}\text{/}\text{Mcl-1}\text{]}\text{-}{\text{(}\text{d'}}_{\text{Nox}\text{a}}\text{+}$$

$\text{d'}_{\text{Mcl-1}}\text{)}\text{[}\text{Nox}\text{a/}\text{Mcl-1}\text{]}$

$$\text{(}\text{24}\text{)}{\text{d[}\text{Puma/}\text{Mcl-1}\text{]}}/\text{dt}=\text{k}_{\text{f}\text{12}}\text{[Puma][}\text{Mcl-1}\text{]}\text{-}\text{k}_{\text{r}\text{12}}\text{[Puma/}\text{Mcl-1}\text{]}\text{-} {\text{(}\text{d'}}_{\text{Pum}\text{a}}\text{+}$$

$$\text{d'}_{\text{Mcl-1}}\text{)}\text{[}\text{Pum}\text{a/}\text{Mcl-1}\text{]}$$

${\text{(}\text{25}\text{)}{\text{d[mito\_p53}\text{/}\text{Mcl-1}\text{]}}/\text{dt}\text{=}\text{k}}_{\text{f}\text{13}}\text{[mito\_p53][}\text{Mcl-1}\text{]}{\text{-}\text{k}}_{\text{r}\text{13}}\text{[mito\_p53/}\text{Mcl-1}\text{]}$-

$${\text{(}\text{d'}}_{\text{mito\_p53}}\text{+}\text{d'}_{\text{Mcl-1}}\text{)}\text{[mito\_p53/}\text{Mcl-1}\text{]}$$

${\text{(}\text{26}\text{)}{\text{d[ac\_Bax}\text{/}\text{Mcl-1}\text{]}}/{\text{dt}\text{=}}\text{k}}_{\text{f}\text{14}}\left[ \text{ac\_Bax} \right]\text{[}\text{Mcl-1}\text{] }\text{-}\text{k}_{\text{r}\text{14}}\text{[ac\_Bax/}\text{Mcl-1}\text{]}$-

${\text{(}\text{d'}}_{\text{ac\_Bax}}\text{+}\text{d'}_{\text{Mcl-1}}\text{)}\text{[ac\_Bax/}\text{Mcl-1}\text{]}$

$${\text{(}\text{27}\text{)}{\text{d[}\text{Puma/}\text{Bcl-xl}\text{]}}/{\text{dt}\text{=}}\text{k}}_{\text{f}\text{15}}\text{[Puma][}\text{Bcl-xl}\text{]+}\text{k}_{\text{r}\text{15}}\text{[Puma/}\text{Bcl-xl}\text{]}\text{ -} {\text{(}\text{d'}}_{\text{Pum}\text{a}}\text{+}$$

$\text{d'}_{\text{Bcl-xl}}\text{)}\text{[}\text{Pum}\text{a/}\text{Bcl-xl}\text{]}$

${\text{(}\text{28}\text{)}{\text{d[mito\_p53}\text{/}\text{Bcl-xl}\text{]}}/\text{dt}\text{=}\text{k}}_{\text{f}\text{16}}\text{[mito\_p53][}\text{Bcl-xl}\text{] +}\text{k}_{\text{r}\text{16}}\text{[mito\_p53/}\text{Bcl-xl}\text{]}$-

$${\text{ (}\text{d'}}_{\text{mito\_p53}}\text{+}\text{d'}_{\text{Bcl-xl}}\text{)}\text{[mito\_p53/}\text{Bcl-xl}\text{]}$$

${\text{(}\text{29}\text{)}{\text{d[ac\_Bax}\text{/}\text{Bcl-xl}\text{]}}/{\text{dt}\text{=}}\text{k}}_{\text{f}\text{17}}\left[ \text{ac\_Bax} \right]\text{[}\text{Bcl-xl}\text{]}$-$\text{k}_{\text{r}\text{17}}\text{[ac\_Bax/}\text{Bcl-xl}\text{]}$-

$${\text{ (}\text{d'}}_{\text{ac\_Bax}}\text{+}\text{d'}_{\text{Bcl-xl}}\text{)}\text{[ac\_Bax/}\text{Bcl-xl}\text{]}$$

We first present an overview of the extended network dynamics at two typical DNA damage (etoposide) doses (Fig. S2).And the results are consistent with the experimental observations of ref. [[8](#_ENREF_8)], same as the results of original apoptosis pathway. Likewise, using the DNA damage level as the control parameter, a saddle-node bifurcation was found in this analysis. The transition diagram of saddle-node bifurcations is presented in Fig. S3.

1.4. The parameters in the ODE

The initial conditions are [p53]=0; [pho-p53]=0; [RNA]=0; [MDM2]=0.5; [p53/MDM2]=0; [mono-ub-p53]=0; [mono-ub-p53/MDM2]=0; [poly-ub-p53]=0; [mito-p53]=0; [BAX]=0; [ac-BAX]=0; [Bcl-2]=1; [PUMA]=0; [ac-BAX/Bcl-2]=0; [mito-p53/Bcl-2]=0; [PUMA/Bcl-2]=0; [ac-BAX/ac-BAX]=0; [pre-casp]=0; [caspase]=0. The values, descriptions and sources for all parameters are listed below. Parameter values are chosen within the same scale with available experiments and/or previous models. The concentration unit is “μM” and time unit is “minute”.

| Parameter | value | description | source |
| --- | --- | --- | --- |
|  | $(\text{damage)}\times0.002$ | Damage-dependent degradation rate of Mdm2 | [[9](#_ENREF_9)] |
|  | $(\text{damage)}\times0.01$ | Damage-dependent Phosphorylation rate of p53 | [[10](#_ENREF_10)] |
|  | 0.02 | Generation rate of p53 | [[10](#_ENREF_10)] |
|  | 0.001 | Basal degradation rate of p53 | [[9](#_ENREF_9)] |
|  | 0.001 | Basal degradation rate of pho-p53 | Same as  |
|  | 0.001 | Basal degradation rate of mito-p53 | [[9](#_ENREF_9),[11](#_ENREF_11),[12](#_ENREF_12)] |
|  | 1 | Degradation rate of poly-ubiquited p53 | [[9](#_ENREF_9)] |
|  | 0.00025 | Nuclear-export rate of mono-ubiquited p53 | Estimated from  |
|  | 0.005 | basal generation rate of Mdm2 mRNA | [[10](#_ENREF_10)] |
|  | 0.05 | P53-dependent generation rate of Mdm2 mRNA | [[10](#_ENREF_10)] |
|  | 0.7 | Michaelis constant of p53-dependent Mdm2 transcription | [[10](#_ENREF_10)] |
|  | 0.05 | Degradation rate of Mdm2 mRNA | [[10](#_ENREF_10)] |
|  | 0.01 | Translation rate of Mdm2 | [[10](#_ENREF_10)] |
|  | 0.5 | Dephosphorylation rate of pho-p53 | Estimated from |
|  | 0.5 | Deubiquitination rate of mono-ubiquited p53 | [[13](#_ENREF_13)] |
|  | 0.05 | De-ubiquitination rate of poly-ubiquited p53 | [[13](#_ENREF_13)] |
|  | 4 | Association rate of p53 and Mdm2 | [[14](#_ENREF_14)] |
|  | 0.2 | Dissociation rate of p53/Mdm2 complex | [[14](#_ENREF_14)] |
|  | 0.1 | Rate of p53 ubiquitination by Mdm2 in p53/Mdm2 complex | [[13](#_ENREF_13)] |
|  | 4 | Association rate of mono-ub-p53 and Mdm2 | [[14](#_ENREF_14)] |
|  | 0.2 | Dissociation rate of mono-ub-p53/Mdm2 complex | [[14](#_ENREF_14)] |
|  | 0.5 | Rate of mono-ub-p53 poly-ubiquitination by Mdm2 in mono-ub-p53/Mdm2 complex | [[13](#_ENREF_13)] |
|  | 0.02 | Basal generation rate of Bax | [[15](#_ENREF_15)] |
|  | 0.02 | P53-dependent generation rate of Bax | [[15](#_ENREF_15)] |
|  | 0.3 | Michaelis constant of p53-dependent Bax generation | [[15](#_ENREF_15)] |
|  | 0.017 | Degradation rate of Bax | the half-life of proteins is generally about one hour |
|  | 0.02 | Basal generation rate of Bcl2 | Estimated from  |
|  | 0.02 | P53-dependent generation rate of Bcl2 | Estimated from  |
|  | 0.3 | Michaelis constant of p53-dependent Bcl2 generation | Estimated from  |
|  | 0.017 | Degradation rate of Bcl2 | Same as  |
|  | 0.03 | Basal generation rate of Puma | Estimated from  |
|  | 0.03 | P53-dependent generation rate of Puma | Estimated from  |
|  | 0.4 | Michaelis constant of p53-dependent Puma generation | Estimated from  |
|  | 0.017 | Degradation rate of Puma | Same as  |
|  | 3 | Mito-p53 dependent activation rate of Bax | [[13](#_ENREF_13)] |
|  | 0.2 | Michaelis constant for Mito-p53 dependent activation rate of Bax | [[13](#_ENREF_13)] |
|  | 1 | Caspase dependent activation rate of Bax | [[16](#_ENREF_16)] |
|  | 0.5 | Michaelis constant for caspase dependent activation rate of Bax | [[16](#_ENREF_16)] |
|  | 1 | Deactivation rate of ac-Bax | [[16](#_ENREF_16)] |
|  | 2 | Ac-bax complex dependent activation rate for the downstream event | [[13](#_ENREF_13)] |
|  | 0.5 | Michaelis constant for Ac-bax complex dependent activation for the downstream event | [[13](#_ENREF_13)] |
|  | 0.03 | Generation rate of pre-caspase | [[16](#_ENREF_16)] |
|  | 0.017 | Degradation rate of pre-caspase | Same as  |
|  | 0.017 | Degradation rate of caspase | Same as  |
|  | 0.1 | Association rate of mito-p53 and Bcl2 | [[17](#_ENREF_17)] |
|  | 0.01 | Dissociation rate of mito-p53/Bcl2 complex | [[17](#_ENREF_17)] |
|  | 3 | Association rate of ac-Bax and Bcl2 | [[18](#_ENREF_18),[19](#_ENREF_19)] |
|  | 0.2 | Dissociation rate of ac-Bax/Bcl2 complex | [[18](#_ENREF_18),[19](#_ENREF_19)] |
|  | 3.6 | Association rate of Puma and Bcl2 | [[17](#_ENREF_17)] |
|  | 0.2 | Dissociation rate of Puma/Bcl2 complex | [[17](#_ENREF_17)] |
|  | 0.5 | ac-Bax dimerization rate | [[18](#_ENREF_18)] |
|  | 0.3 | ac-Bax complex undimerization rate | [[18](#_ENREF_18)] |
|  | 0.017 | Degradation rate of Bcl2 in the complex | Same as  |
|  | 0.01 | Degradation rate of Bax in the complex | Similar as |
|  | 0.001 | Degradation rate of mito-p53 in the complex | Same as  |
|  | 0.017 | Degradation rate of Puma in the complex | Same as  |
| $\text{g}_{{\text{c}\text{-}}_{\text{Noxa}}}$ | 0.01 | Basal generation rate of Noxa | Estimated from  $\text{g}_{{\text{c}\text{-}}_{\mathrm{Bax}}}$ |
| V5 | 0.001 | P53-dependent generation rate of Noxa | Estimated from V2 |
| j5 | 0.5 | Michaelis constant of p53-dependent Noxa generation | Estimated from j2 |
| $\text{d}_{\text{Noxa}}$ | 0.017 | Degradation rate of Noxa | Same as  $\text{d}_{\mathrm{Bax}}$ |
| $\text{g}_{\text{c\_}\text{Mcl-1}}$ | 0.02 | Generation rate of Mcl-1 | Estimated from  $\text{g}_{{\text{c}\text{-}}_{\mathrm{Bax}}}$ |
| $\text{d}_{\text{Mcl-1}}$ | 0.017 | Degradation rate of Mcl-1 | Same as  $\text{d}_{\mathrm{Bax}}$ |
| $\text{g}_{\text{c\_}\text{Bcl-xl}}$ | 0.01 | Generation rate of Bcl-xl | Estimated from  $\text{g}_{{\text{c}\text{-}}_{\mathrm{Bax}}}$ |
| $\text{d}_{\text{Bcl-xl}}$ | 0.017 | Degradation rate of Bxl-xl | Same as  $\text{d}_{\mathrm{Bax}}$ |
| $\text{k}_{\text{f}\text{11}}$ | 3.6 | Association rate of Noxa and Mcl-1 | [[20](#_ENREF_20)] |
| $\text{k}_{\text{r}\text{11}}$ | 0.2 | Dissociation rate of Noxa/Mcl-1 complex | [[20](#_ENREF_20)] |
| $\text{k}_{\text{f}\text{12}}$ | 3.6 | Association rate of Puma and Mcl-1 | Estimated from $\text{k}_{\text{f}\text{9}}$ |
| $\text{k}_{\text{r}\text{12}}$ | 0.2 | Dissociation rate of Puma/Mcl-1 complex | Estimated from  $\text{k}_{\text{r}\text{9}}$ |
| $\text{k}_{\text{f}\text{13}}$ | 0.1 | Association rate of mito-p53 and Mcl-1 | Estimated from  $\text{k}_{\text{f}\text{7}}$ |
| $\text{k}_{\text{r}\text{13}}$ | 0.01 | Dissociation rate of mito-p53/Mcl-1 complex | Estimated from  $\text{k}_{\text{r}\text{7}}$ |
| $\text{k}_{\text{f}\text{14}}$ | 3 | Association rate of ac-Bax and Mcl-1 | [[21](#_ENREF_21)] |
| $\text{k}_{\text{r}\text{14}}$ | 0.2 | Dissociation rate of ac-Bax/Mcl-1 complex | [[21](#_ENREF_21)] |
| $\text{k}_{\text{f}\text{15}}$ | 3.6 | Association rate of Puma and Bcl-xl | [[17](#_ENREF_17)] |
| $\text{k}_{\text{r}\text{15}}$ | 0.2 | Dissociation rate of Puma/Bcl-xl complex | [[17](#_ENREF_17)] |
| $\text{k}_{\text{f}\text{16}}$ | 0.1 | Association rate of mito-p53 and Bcl-xl | [[18](#_ENREF_18)] |
| $\text{k}_{\text{r}\text{16}}$ | 0.01 | Dissociation rate of mito-p53/Bcl-xl complex | [[18](#_ENREF_18)] |
| $\text{k}_{\text{f}\text{17}}$ | 3 | Association rate of ac-Bax and Bcl-xl | [[18](#_ENREF_18),[19](#_ENREF_19)] |
| $\text{k}_{\text{r}\text{17}}$ | 0.2 | Dissociation rate of ac-Bax/ Bcl-xl complex | [[18](#_ENREF_18),[19](#_ENREF_19)] |
| $\text{d'}_{\text{Nox}\text{a}}$ | 0.017 | Degradation rate of Puma in the complex | ${\mathrm{Same}\mathrm{as}\text{d}}_{\mathrm{Bax}}$ |
| $\text{d'}_{\text{Mcl-1}}$ | 0.017 | Degradation rate of Puma in the complex | $\mathrm{Same}\mathrm{as}\text{d}_{\mathrm{Bax}}$ |
| $\text{d'}_{\text{Bcl-xl}}$ | 0.017 | Degradation rate of Puma in the complex | ${\mathrm{Same}\mathrm{as}\text{d}}_{\mathrm{Bax}}$ |

**2. The corresponding of sensitive parameters and gene mutations**

2.1. Corresponding rules

Based on the knowledge of the biochemical reactions and gene expression, this paper will get three forms of gene mutations: mutation, amplification and deletion. The correspondences of mutations with specific parameters in ordinary differential equation (ODE) model are established by the following rules:

(1) In the production of protein A, we believe that the generation rate increased corresponding to gene A amplification, the reduced corresponding to the A gene deletion. In the case of transcription factor T or transcription inhibitor R, the change of generation rate may also be related to the changes in the catalytic activity of the T-protein with the mutation of T gene; the parameters in the denominator corresponds to the T or R and DNA binding capacity, thus it is related to the gene T or R mutation.

(2) In general, the degradation of protein A is related with A gene mutation. When the degradation of protein A is under the regulation of another protein B, the relevant parameters is also related with the B gene mutation.

(3) In the case of the combination of several proteins and their complexes dissociation process, parameter changes are likely related to the each monomer protein gene mutation.

(4) Enzymatic reaction rate and the parameters on the denominator are likely to be relevant with substrate and enzyme gene mutation.

2.2. The choice of spectrum of cancer gene mutation

To identify the corresponding relationship between the model parameters changes and the spectrum of cancer gene mutation, we choose the skin cancer mutated genes from the Catalogue of Somatic Mutations in Cancers (COSMIC)[[22](#_ENREF_22)] (http://www.sanger.ac.uk/genetics/CGP/cosmic), and glioblastoma multiforme mutated genes by TCGA [[23](#_ENREF_23)] and from the ‘‘CAN-genes’’ by Parsons et al. [[24](#_ENREF_24)] as the spectrum of cancer gene mutation. The TCGA project has cataloged somatic mutations and recurrent copy number alterations in 91 glioblastoma multiforme cases [[23](#_ENREF_23)], and the ‘‘CAN-genes’’ involved the frequently mutated genes sequencing of 22 glioblastoma multiforme samples. The specific corresponding of parameters and gene mutations present in the Table S1.

**3. Parameter Sensitivity Analysis and the correspondence of the parameter sensitivity and mutation genes**

3.1 Parameters sensitivity analysis of the saddle node bifurcation

We do parameters sensitivity analysis of saddle node bifurcation in our model, by 1.2-fold multiplication and division change of a single parameter for all the 54 parameters and recording the percentage change of the critical point of bifurcation and steady-state concentrations of caspase3. (See Fig. 4, Table S2, Table S3)

3.2 Parameters sensitivity analysis of Hopf bifurcation

We also conduct parameter sensitivity analysis of the Hopf bifurcation of nuclear p53 as a function of the DNA damage level [[1](#_ENREF_1)] and found a high correlation between the spectrum of parameter sensitivity and the oncogenic mutation spectrum (see Fig. S1A, Fig. S1B). This may indicate that the nuclear p53 oscillation may play a crucial role to safeguard cell from turning to cancerous

3.3 Parameters sensitivity analysis of the saddle node bifurcation in extended model

In extended model, we conducted parameters sensitivity analysis of saddle node bifurcation for all the 79 parameters and recording the percentage change of the critical point of bifurcation and steady-state concentrations of caspase3 (Fig. S3). And the results of parameters sensitivity analysis and the correspondence of the parameter sensitivity and mutation genes are shown in Fig.8. In Fig. 18, we found the sensitive parameters in the extended pathway are almost the same as that in the original pathway. The 15 parameters (yellow in Fig. 4A) which lead to the largest changes in the bifurcation points is also the 15 largest sensitive parameters to the bifurcation points in the extended pathway (yellow in Fig. 8A). The difference is that 3 more sensitive parameters in the added 25 parameters are found in the extended pathway. Similar with results of the original pathway (Fig. 5A), we found all sensitive parameters to the bifurcation point correspond to the some mutations (mutation hot spots) of skin cancer and glioblastoma multiforme in the extended pathway (Fig. 8C). Besides, we compared all 18 parameters which have a strong effect on the critical bifurcation points with their effect on the level of steady-state of caspase3 (the blue column Fig. 8C). Similarly, the two parameters (magenta in Fig. 4B) which lead to the largest changes in the steady-state concentration of caspase3 are also the two largest sensitive parameters to the steady-state concentration in the extended pathway (magenta in Fig.8B). We also calculated the changes of critical point with the changes of those the parameters (Fig. S4). And all the results in the extended pathway are almost the same as that in the original pathway.

**References**

1. Li Z, Ni M, Li J, Zhang Y, Ouyang Q, et al. (2010) Decision making of the p53 network: Death by integration. Journal of theoretical biology 10.1016/j.jtbi.2010.11.041.

2. Olsson A, Manzl C, Strasser A, Villunger A (2007) How important are post-translational modifications in p53 for selectivity in target-gene transcription and tumour suppression? Cell death and differentiation 14: 1561-1575.

3. Bagci EZ, Vodovotz Y, Billiar TR, Ermentrout GB, Bahar I (2006) Bistability in apoptosis: roles of bax, bcl-2, and mitochondrial permeability transition pores. Biophys J 90: 1546-1559.

4. Rehm M, Huber HJ, Dussmann H, Prehn JH (2006) Systems analysis of effector caspase activation and its control by X-linked inhibitor of apoptosis protein. Embo J 25: 4338-4349.

5. Strom E, Sathe S, Komarov PG, Chernova OB, Pavlovska I, et al. (2006) Small-molecule inhibitor of p53 binding to mitochondria protects mice from gamma radiation. Nature chemical biology 2: 474-479.

6. Letai AG (2008) Diagnosing and exploiting cancer's addiction to blocks in apoptosis. Nature reviews Cancer 8: 121-132.

7. Chen L, Willis SN, Wei A, Smith BJ, Fletcher JI, et al. (2005) Differential targeting of prosurvival Bcl-2 proteins by their BH3-only ligands allows complementary apoptotic function. Molecular cell 17: 393-403.

8. Chen X, Chen J, Gan S, Guan H, Zhou Y, et al. (2013) DNA damage strength modulates a bimodal switch of p53 dynamics for cell-fate control. BMC biology 11: 73.

9. Ciliberto A, Novak B, Tyson JJ (2005) Steady states and oscillations in the p53/Mdm2 network. Cell Cycle 4: 488-493.

10. Ma L, Wagner J, Rice JJ, Hu W, Levine AJ, et al. (2005) A plausible model for the digital response of p53 to DNA damage. Proceedings of the National Academy of Sciences of the United States of America 102: 14266-14271.

11. Blagosklonny MV (2000) p53 from complexity to simplicity: mutant p53 stabilization, gain-of-function, and dominant-negative effect. Faseb J 14: 1901-1907.

12. Tang M, Wahl GM, Nister M (2006) Explaining the biological activity of transactivation-deficient p53 variants. Nat Genet 38: 395-396; author reply 396-397.

13. Aldridge BB, Burke JM, Lauffenburger DA, Sorger PK (2006) Physicochemical modelling of cell signalling pathways. Nat Cell Biol 8: 1195-1203.

14. Sakaguchi K, Saito S, Higashimoto Y, Roy S, Anderson CW, et al. (2000) Damage-mediated phosphorylation of human p53 threonine 18 through a cascade mediated by a casein 1-like kinase. Effect on Mdm2 binding. J Biol Chem 275: 9278-9283.

15. Chipuk JE, Kuwana T, Bouchier-Hayes L, Droin NM, Newmeyer DD, et al. (2004) Direct activation of Bax by p53 mediates mitochondrial membrane permeabilization and apoptosis. Science 303: 1010-1014.

16. Hua F, Cornejo MG, Cardone MH, Stokes CL, Lauffenburger DA (2005) Effects of Bcl-2 levels on Fas signaling-induced caspase-3 activation: molecular genetic tests of computational model predictions. J Immunol 175: 985-995.

17. Chipuk JE, Bouchier-Hayes L, Kuwana T, Newmeyer DD, Green DR (2005) PUMA couples the nuclear and cytoplasmic proapoptotic function of p53. Science 309: 1732-1732.

18. Tan YJ, Beerheide W, Ting AE (1999) Biophysical characterization of the oligomeric state of Bax and its complex formation with Bcl-XL. Biochem Biophys Res Commun 255: 334-339.

19. Sensitive C, Dlugosz PJ, Billen LP, Annis MG, Zhu W, et al. (2006) Top (Index), File: 16642033. pdf. The EMBO Journal 25: 2287-2287.

20. Oda E, Ohki R, Murasawa H, Nemoto J, Shibue T, et al. (2000) Noxa, a BH3-only member of the Bcl-2 family and candidate mediator of p53-induced apoptosis. Science 288: 1053-1058.

21. Willis SN, Chen L, Dewson G, Wei A, Naik E, et al. (2005) Proapoptotic Bak is sequestered by Mcl-1 and Bcl-xL, but not Bcl-2, until displaced by BH3-only proteins. Genes & development 19: 1294-1305.

22. Forbes SA, Bhamra G, Bamford S, Dawson E, Kok C, et al. (2008) The Catalogue of Somatic Mutations in Cancer (COSMIC). Current protocols in human genetics / editorial board, Jonathan L Haines [et al] Chapter 10: Unit 10 11.

23. (2008) Comprehensive genomic characterization defines human glioblastoma genes and core pathways. Nature 455: 1061-1068.

24. Parsons DW, Jones S, Zhang X, Lin JC, Leary RJ, et al. (2008) An integrated genomic analysis of human glioblastoma multiforme. Science 321: 1807-1812.
